# Peptide Supramolecular Assembly‐Instructed In Situ Self‐Aggregation for Stratified Targeting Sonodynamic Therapy Enhancement of AIE Luminogens

**DOI:** 10.1002/advs.202204989

**Published:** 2022-12-09

**Authors:** Weixi Jiang, Chen Cheng, Xiaoling Qiu, Li Chen, Xun Guo, Yuanli Luo, Jingxue Wang, Junrui Wang, Zhuoyan Xie, Pan Li, Zhigang Wang, Haitao Ran, Zhiyi Zhou, Jianli Ren

**Affiliations:** ^1^ Department of Ultrasound and Chongqing Key Laboratory of Ultrasound Molecular Imaging the Second Affiliated Hospital of Chongqing Medical University No.74 Linjiang Rd, Yuzhong District Chongqing 400010 P. R. China; ^2^ Department of Ultrasound Bishan Hospital of Chongqing Bishan Hospital of Chongqing Medical University No. 9 Shuangxing Avenue, Biquan Street, Bishan District Chongqing 402760 P. R. China; ^3^ Department of Intensive Care Unit the Second Affiliated Hospital of Chongqing Medical University No.74 Linjiang Rd, Yuzhong District Chongqing 400010 P. R. China; ^4^ Department of Radiology the Second Affiliated Hospital of Chongqing Medical University No.74 Linjiang Rd, Yuzhong District Chongqing 400010 P. R. China; ^5^ Department of Ultrasound Chongqing General Hospital NO. 118 Xingguang Avenue, Liangjiang New Area Chongqing 401147 P. R. China; ^6^ Department of General practice Chongqing General Hospital NO. 118 Xingguang Avenue, Liangjiang New Area Chongqing 401147 P. R. China

**Keywords:** aggregation‐induced emission, in situ self‐aggregation, peptide‐based supramolecular self‐assembly, sonodynamic therapy, stratified targeting

## Abstract

The emergence of aggregation‐induced emission luminogens (AIEgens) has attracted substantial scientific attention. However, their antitumor efficacy in photodynamic therapy (PDT) is significantly restricted by the poor water solubility and limited treatment depth. Therefore, a novel AIEgens‐involved therapeutic platform with good permeability and bioavailability is urgently required. Herein, supramolecular chemistry is combined with the AIEgen bis‐pyrene (BP) to construct a peptide–AIEgen hybrid nanosystem (PAHN). After intravenous injection, the versatile nanoplatform not only improved the hydrophilicity of BP but also achieved stratified targeting from tumor to mitochondrial and induced mitochondrial dysfunction, thus activating caspase‐3 upregulation. Then, sonodynamic therapy (SDT), an alternative modality with high tissue penetrability, is performed to evoke reactive oxygen species (ROS) generation for BP. More importantly, since the hydrophilic shell is separated from the nanosystem by the specific cleavage of caspase‐3, the resulting decrease in hydrophilicity induced tight self‐aggregation of PAHN residues in situ, further allowing more absorbed energy to be used for ROS generation under ultrasound irradiation and enhancing SDT efficacy. Moreover, severe oxidative stress resulting from ROS imbalance in the mitochondria initiates the immunogenic cell death process, thus evoking antitumor immunogenicity. This PAHN provides prospective ideas into AIE‐involved antitumor therapy and design of peptide‐AIEgens hybrids.

## Graphical abstract

Inspired by the characteristics of aggregation‐induced emission luminogens (AIEgens), a peptide‐AIEgen hybrid nanosystem (PAHN) with a unique strategy of “stratified targeting” and “in situ self‐aggregation” is designed for synergistic enhanced sonodynamic therapy (SDT). The specific cleavage of caspase‐3 induces tight self‐aggregation of PAHN, further allowing more absorbed energy used for reactive oxygen species generation and enhancing mitochondria‐mediated oxidative stress.

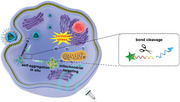

## 1 Introduction

Traditional photosensitizers (PSs) can easily aggregate upon overloading in a matrix, resulting in quenched fluorescence and reduced reactive oxygen species (ROS) generation due to the aggregation‐caused quenching (ACQ) effect.^[^ ^1^ ^]^ To overcome this disadvantage, novel fluorophores with the property of aggregation‐induced emission (AIE) have been developed. Due to the propeller‐shaped or twisted molecular structures, intramolecular motion of AIE luminogens (AIEgens) will be restricted in the aggregated or solid state, thereby preventing excitons from undergoing nonradiative decay after excitation. Thus, the energy level difference between singlet (S_0_) and triplet (T_1_) excited states decreases and intersystem crossing (ISC) increases, so that absorbed excitation energy can be used to achieve “weak–strong” fluorescence emission and ROS generation as much as possible.^[^ ^2^, ^3^ ^]^ This makes AIE‐based PSs promising for biomedical applications, such as tumor theranostics,^[^ ^4^ ^]^ pathogen elimination^[^ ^5^ ^]^ and cell imaging.^[^ ^6^ ^]^ However, even though AIEgens have been rapidly applied in the field of photodynamic therapy (PDT), most of these PSs have short absorption wavelengths and they can only be excited by light below 700 nm, which greatly restricts their treatment depth in tumor therapy.^[^ ^7^ ^]^ In addition, the hydrophobicity and poor tumor‐targeting ability of AIEgens further impair their bioavailability. Therefore, it is crucial to find a novel therapeutic platform with good tissue permeability and bioavailability to expand the clinical application of AIEgens.

As an alternative approach to tumor destruction, sonodynamic therapy (SDT), which is characterized by high therapeutic efficiency and minimal invasiveness, has been applied in cancer therapeutics.^[^ ^8^ ^]^ Similar to the antineoplastic mechanism of PDT, sonosensitizers undergo energy transition under the irradiation of ultrasound (US) energy and finally generate abundant ROS, especially singlet oxygen (^1^O_2_), causing oxidative damage to cancer cells. More importantly, US waves possess high tissue penetration ability, which makes SDT an emerging modality for AIE‐involved antitumor therapy. To date, the research on exploring the SDT efficiency of AIEgens and proposing practical strategies to enhance AIEgens‐mediated SDT is still in its infancy. Peptide‐based supramolecular assemblies have been proven to be promising in the construction of biological materials, which usually consist of several modularized subunits via noncovalent interactions, such as hydrophobic/hydrogen bonding, *π*–*π* stacking and electrostatic interactions. Notably, peptide‐based supramolecular assemblies with unique designs not only stabilize the loaded cargoes but also retain the multiple capacities of the peptide itself, including targeting capability,^[^ ^9^ ^]^ stimuli responsiveness^[^ ^10^ ^]^ and controllable structural transformation.^[^ ^11^ ^]^ This technology has been broadly applied in small molecule delivery.^[^ ^12^ ^]^

**Scheme 1:**
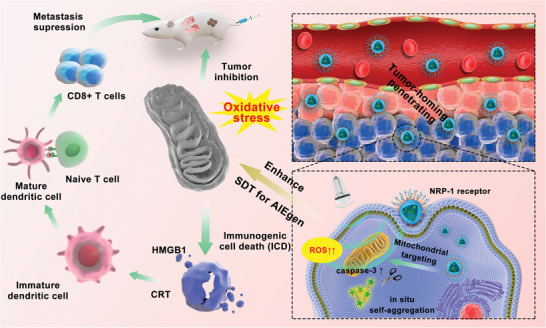
Schematic illustration of the PAHN‐mediated SDT and antitumor immunity effect via mitochondrial oxidative stress.

Inspired by the characteristics of AIEgens in the aggregated state, herein, we creatively combined AIEgen bis‐pyrene (BP) with peptide‐based supramolecular assemblies to construct a peptide–AIEgen hybrid nanosystem (PAHN) for enhanced SDT‐mediated tumor inhibition, as presented in **Scheme** **1**. PAHN‐1 (BP‐CFFFVLKLAKLAKDEVDAKRGARSTA) consists of four functional domains, including: i) a tumor homing and penetrating motif (AKRGARSTA) that can specifically recognize neuropilin‐1 (NRP‐1)‐expressing solid tumors;^[^ ^13^, ^14^ ^]^ ii) a caspase‐3‐cleavable linker (DEVD);^[^ ^15^ ^]^ iii) a self‐assembly motif (CFFFVLKLAKLAK) with the capacity for mitochondrial targeting and perturbation;^[^ ^16^ ^]^ and iv) an ROS generator (BP) with AIE effect.^[^ ^17^ ^]^ In aqueous solution, PAHN‐1 self‐assembles into spherical nanoparticles (NPs) via a rapid precipitation mechanism, in which the BP and FFVLK sequences form a hydrophobic core and the AKRGARSTA peptide forms a hydrophilic shell.^[^ ^18^ ^]^ The well‐ordered nanostructure not only prevents the rapid clearance of small‐molecule drugs in vivo but also confers desirable water solubility on the highly hydrophobic AIEgen. After the intravenous injection of PAHN‐1, the tumor recognition motif AKRGARSTA endows the nanosystem with extraordinary tumor homing and penetration behavior, allowing AIEgen to preferentially accumulate in the tumor site and deeply penetrate into cells by spanning the vessel–stroma barrier. After cell internalization, the second motif CFFFVLKLAKLAK, which contains a positively charged KLA sequence drives PAHN‐1 to target the mitochondria and then upregulates active caspase‐3 expression via mitochondrial dysfunction.^[^ ^19^ ^]^ More impressively, upregulated caspase‐3 then cleaves the enzyme‐responsive linker (DEVD), disconnecting the hydrophilic shell from PAHN‐1, which then induces tight self‐aggregation of PAHN residues in situ due to the exposure of hydrophobic segments.^[^ ^20^ ^]^ In this situation, BP allows more absorbed energy to be used for ROS production under US irradiation than that in free molecule form because a more tightly aggregated state further strengthens the photoactivity of AIEgens through RIM.^[^ ^2^ ^]^ This well‐designed nanosystem not only realizes targeting and penetrating destruction from the tumor to mitochondria but also undergoes in situ self‐aggregation, ultimately resulting in a significant SDT enhancement. Furthermore, because of the severe oxidative stress resulting from ROS imbalance in mitochondria, PAHN‐induced SDT initiates immunogenic cell death (ICD), which can improve the low immunogenicity of “cold” tumors to obtain “hot” tumors by releasing numerous damage‐associated molecular patterns (DAMPs).^[^ ^21^ ^]^ These vaccine‐like effects could induce dendritic cell (DC) maturation and then present antigens to stimulate the proliferation of cytotoxic T lymphocytes (CTLs) and generate inflammatory cytokines to inhibit tumor invasion and metastasis.^[^ ^22^ ^]^

In conclusion, this study demonstrated that BP with AIE properties can be applied as a promising sonosensitizer to kill cancer cells. Moreover, we demonstrated for the first time that peptide‐based supramolecular chemistry strongly enhances the therapeutic efficacy of AIEgens‐mediated SDT by implementing the strategy of “stratified targeting” and “stimuli‐responsive self‐aggregation in situ.” This PAHN expands the potential of AIEgens in the clinical application while providing prospective ideas for both design and therapy of peptide–AIEgen hybrids.

## 2 Results and Discussion

### 2.1 Characterization of the PAHN

**Figure 1:**
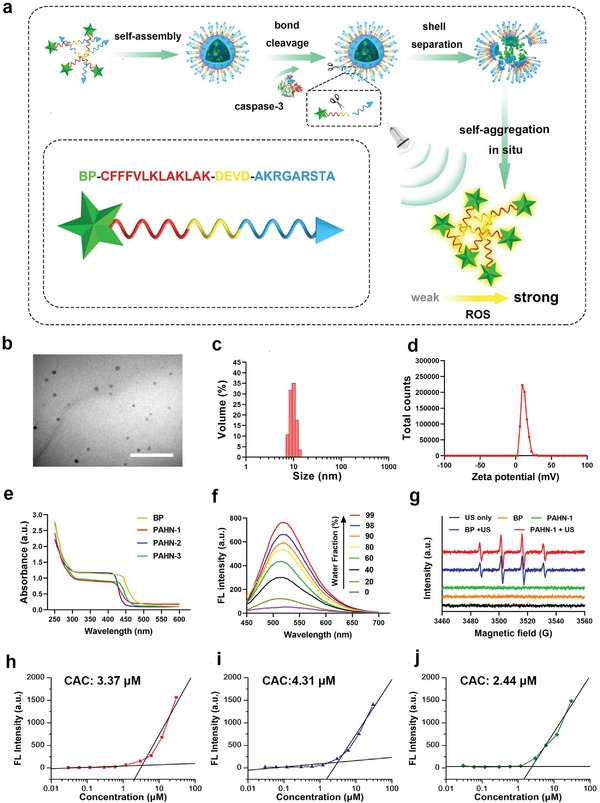
a) Schematic diagram of the formation and disassembling of PAHN. b) TEM images of PAHN‐1. The scale bar is 0.1 µm. c) Size distribution and d) zeta potentials of PAHN‐1. e) UV spectrum of BP, PAHN‐1, PAHN‐2, PAHN‐3. f) Fluorescence intensity changes of PAHN‐1 in the conditions of different water fractions. g) ESR spectrum of BP and PAHN‐1 with or without US irradiation after adding with DMPO. h–j) CAC detection of PAHN‐1, PAHN‐2, and PAHN‐3.

The design of smart peptide–AIEgens biomaterials is beneficial for AIE‐based antitumor therapy. To this end, the PAHN‐1 (BP‐CFFFVLKLAKLAK‐DEVD‐AKRGARSTA) was synthesized through the standard Fmoc solid‐phase peptide synthesis (SPPS) and the amide condensation method (**Figure** **1**a).^[^ ^23^ ^]^ The molecular weight of PAHN‐1 characterized by high‐resolution mass spectrometry (HRMS) was comparable to that of the target polypeptide and BP (Figure S1b, Supporting Information). High‐performance liquid chromatography (HPLC) revealed a high purity of PAHN‐1 up to 96% (Figure S1c, Supporting Information). In addition, comparing the peaks of PAHN‐1 in proton nuclear magnetic resonance spectroscopy (^1^HNMR) with those of the naked peptide and BP also indicated the successful conjugation of the peptide with the AIEgen (Figure S4, Supporting Information). Moreover, to systematically elucidate the peptide function‐related antitumor behavior, two negative control peptide–AIEgen hybrids, BP‐CFFFVLKLAKLAK‐DEVD‐AKDGAESHH (named PAHN‐2) and BP‐CFFFVLKKK‐DEVD‐AKRGARSTA (named PAHN‐3) were also synthesized and characterized by HRMS and HPLC (Figures S2 and S3, Supporting Information). Transmission electron microscopy (TEM) showed that PAHN‐1, PAHN‐2, and PAHN‐3 had spherical morphologies with nanoscale dimensions (Figure 1b and Figure S5, Supporting Information). Dynamic light scattering (DLS) revealed that the diameters of nanosystems 1–3 were 13.05 ± 3.87, 14.34 ± 7.4, and 12.58 ± 0.79 nm, respectively (Figure 1c and Figure S5, Supporting Information). In particular, nonsignificant size changes were observed in different physiological conditions such as deionized water, culture medium, phosphate buffer (PBS) and saline, revealing the good water solubility and stability of this nanosystem (Figure S6, Supporting Information). Additionally, the zeta potentials were found to be 12.86 ± 0.97, −4.05 ± 0.08, and 24.83 ± 2.21 mV, respectively. The significant variation in zeta potential among these NPs is probably due to the different peptide components, especially tumor homing‐penetrating peptide (THPP), which usually has a positive charge (Figure 1d and Figure S7, Supporting Information).^[^ ^24^ ^]^ UV spectroscopy revealed that PAHN‐1, PAHN‐2 and PAHN‐3 had broad absorption peaks (≈250–450 nm), comparable to the free BP molecule, as shown in Figure 1e, again suggesting the successful conjugation between the peptide and AIEgen. To demonstrate the AIE property of PAHN, the fluorescences of nanosystems 1–3 in dimethyl sulfoxide (DMSO)–water mixtures were detected. The results showed that weak signals for PAHN‐1, PAHN‐2, and PAHN‐3 in the 100% DMSO because there was no aggregation occurring in a lipophilic environment. However, the fluorescence intensity gradually increased in the emission peaks at 525 nm as the proportion of water increased, and reached fifteenfold at a 99% water fraction compared to its initial intensity, which suggested that the classical AIE effect was maintained (Figure 1f and Figure S8, Supporting Information). To further confirm the self‐assembling behavior of each nanosystem, we measured their critical aggregation concentration (CAC) using a Nile Red (NR) probe. The CAC values of PAHN‐1, 2 and 3 were calculated to be 3.37, 4.31 and 2.44 µm, respectively (Figure 1h–j). Besides, electron spin resonance (ESR) technique was used to verify ROS production performance of PAHN. As shown in Figure 1g, a typical 1:2:2:1 quaternate signal of the 5,5‐dimethyl‐1‐pyrroline‐*N*‐oxide (DMPO), which was used to trap hydroxyl radical (•OH) and form relatively long‐lived radical adducts for detection, was observed after applying US irradiation on BP and PAHN‐1 solutions, however, such a noticeable characteristic signal was not observed in the PBS with US irradiation and solution of BP and PAHN‐1 without US irradiation, thereby indicating that a BP and BP‐involved nanosystem could generate ROS when excited by US.

### 2.2 Tumor Homing and Penetrating Performance

**Figure 2:**
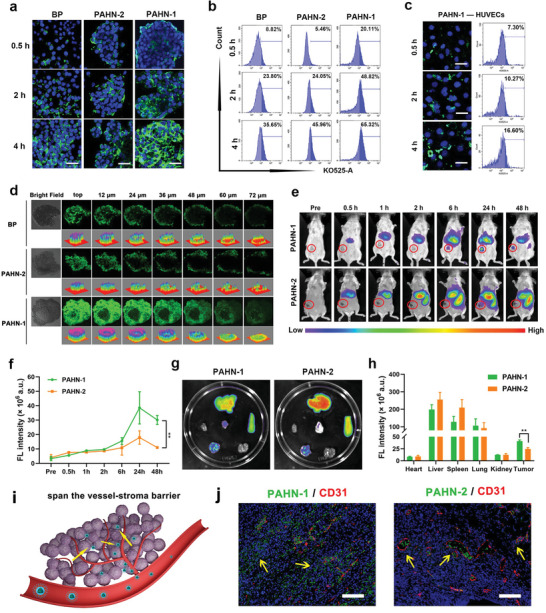
a) Confocal images and b) flow cytometry analysis of the 4T1 cells coincubated with BP, PAHN‐1 and PAHN‐2 for 0.5, 2 and 4 h. The scale bars are 25 µm. c) Confocal images and flow cytometry analysis of the HUVECs after incubating with BP, PAHN‐1 and PAHN‐2 for 0.5, 2, and 4 h. The scale bars are 25 µm. d) Multiple level scan for the penetration analysis of BP, PAHN‐1, and PAHN‐2. e) In vivo fluorescence images and f) corresponding fluorescence intensity of tumor site in 4T1 tumor‐bearing mice after intravenous injection of PAHN‐1 and PAHN‐2 at different time intervals. g) Ex vivo fluorescence images and h) corresponding fluorescence intensity of tumor and major organs 48 h postinjection of PAHN‐1 and PAHN‐2. i) Illustration of vessel–stroma barrier penetration by THPP. j) The localization of PAHN‐2 and PAHN‐1 in the tumor tissue. Red fluorescence represents microvessels stained by CD31. The scale bars are 100 µm. Data are presented as mean ± SD, *n* = 3 per group.

The introduction of the THPP motif accomplishes two goals: improving the biocompatibility of BP in blood circulation and conferring the desirable tumor targeting and penetrating ability. AKRGARSTA is a newly identified THPP, which contains a tumor‐homing domain that can specifically bind to the NRP receptor in malignant tumor, and Cend‐R domain that can promote tissue penetration of nanoplatforms. To verify this effect, the intracellular behavior of the nanosystem was visualized by confocal laser scanning microscopy (CLSM). After 0.5, 2 and 4 h of coincubation of 4T1 breast cancer cell lines with PAHN‐1, PAHN‐2 and free BP, the green fluorescence signal gradually accumulated around the cytomembrane of NRP‐1‐overexpressing 4T1 cells. Additionally, the strongest fluorescence intensity was observed in the PAHN‐1 group after 4 h of incubation compared with that in the PAHN‐2 and free BP groups, indicating a higher cell internalization of the nanosystem with the THPP sequence (**Figure** **2**a). As a control, PAHN‐1 was also incubated with an NRP‐1 receptor‐negative cell line (human umbilical vein endothelial cells, HUVECs). The results showed that the accumulation of PAHN‐1 was markedly lower in HUVECs than in 4T1 cells (Figure 2c). The quantitative analysis of tumor targeting behavior was conducted by flow cytometry. In accordance with the CLSM images, the PAHN‐1 group had the strongest fluorescence intensity (Figure 2b). To better compare the penetration ability of PAHN‐1, PAHN‐2 and free BP, a 3D tumor spheroid model was successfully formed to imitate the in vivo status of the tumor. After 4 h of coincubation, green fluorescence emitted from PAHN‐1 was more clearly distributed in the tumor spheroids and extended more than 70 µm from the top of the tumor spheroids. In contrast, PAHN‐2 and BP adhered only to the margin of the tumor spheroid. (Figure 2d). The penetration depth of PAHN‐1 reached 27.61 µm, which was twice those of PAHN‐2 and BP, which had penetration depths of only 12.82 and 10.12 µm, respectively (Figure S9, Supporting Information). To determine the in vivo targeting and distribution of the self‐assembled nanosystem, fluorescence imaging was conducted at predetermined time intervals. The results showed that both PAHN‐1 and PAHN‐2 gradually accumulated in the tumor site of 4T1 tumor‐bearing mice at 6 h postinjection. Notably, the fluorescence signal of the tumor region was much higher in PAHN‐1‐treated mice than in PAHN‐2‐treated mice and reached its maximum at 24 h. Moreover PAHN‐1 exhibited longer retention in the tumor region than the PAHN‐2 group, as shown by analysis at 48 h postinjection (Figure 2e,f). Correspondingly, the ex vivo images of tumors and major organs (heart, liver, spleen, lung, and kidney) harvested from mice showed that without the targeting ability, PAHN‐2 tended to accumulate in the liver and spleen because of phagocytosis by the reticuloendothelial system (RES); however, accumulation of the PAHN‐1 in the liver and spleen was less. In particular, a relatively greater tumor accumulation was observed in PAHN‐1 group (Figure 2g,h). These results indicated that PAHN‐1 exhibited more effective targeting and longer retention. Surface‐to‐core penetration of nanodrugs after arriving at tumor sites is a longstanding problem in the delivery process due to the stroma barrier and vascular endothelial barrier in the solid tumor.^[^ ^14^ ^]^ THPP is reported to be capable of actively passing through the tumor barrier via the Cend‐R motif (Figure 2i). To further determine the capacity of barrier‐crossing of PAHN in the tumor, tissue slices were prepared. Tumor cells and microvessels in tissue were stained using DAPI and CD31, respectively. The immunofluorescence results demonstrated that the fluorescence of PAHN‐2 was primarily trapped in the tumor vasculature with slight extravasation. Interestingly, PAHN‐1 was widely distributed in the extravascular tumor tissue and reached a deeper area (Figure 2j). Owing to the satisfactory targeting and penetrating performance, we speculated that the versatile nanosystem could facilitate the AIEgens into the core of the tumor, possibly favoring SDT enhancement.

### 2.3 Mitochondrial Targeting and Dysfunction

**Figure 3:**
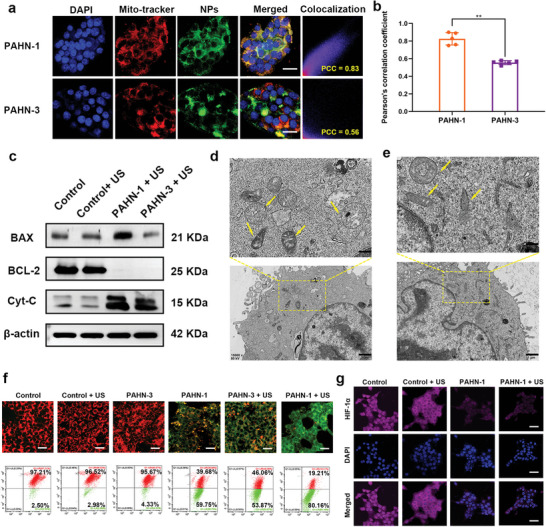
a) The colocalization of mitochondria and NPs in 4T1 cells after coincubation with PAHN‐1 and PAHN‐3. Co‐location scatterplots of PAHN‐1 and PAHN‐3 versus mitochondria are analyzed by Image J. The scale bars are 25 µm. b) Pearson's correlation coefficient (PCC) values of PAHN‐1 and PAHN‐3 versus mitochondria after 6 h coincubation, *n* = 5 per group. c) Western blotting analysis of mitochondria‐related apoptotic pathway after different treatments. Bio‐TEM images of 4T1 cells d) before and e) after PAHN‐1 + US treatment. The yellow arrows indicate mitochondria in a physical or impaired condition. f) Intracellular MMP changes after different treatments analyzed by CLSM and flow cytometry. The scale bars are 25 µm. g) Intracellular hypoxic condition after different treatments. The scale bars are 50 µm.

Precise design strategies in nanomedicine have enabled cancer therapy to expand beyond tissue and cell targeting to organelle targeting. It would be optimal to deliver sonosensitizers not merely into tumor cells, but specifically into those critical subcellular organelles that are most sensitive to ROS, as the diffusion distance (20 nm) and half‐life (40 ns) of ROS are relatively short.^[^ ^25^ ^]^ Because of their crucial role in maintaining metabolic homeostasis in cells, mitochondria are considered important targets for antitumor therapy. Directly ROS generation in the mitochondria could lead to amplified oxidative stress and trigger marked cell apoptosis of tumor cells; therefore, the mitochondrial targeting sequence KLAKLAK was incorporated into the self‐assembly motif. We first compared the mitochondrial targeting efficacy of PAHN‐1 and PAHN‐3 (without a mitochondrial targeting sequence) by observing the colocalization of mitochondria and AIEgens. The results showed a higher overlap between the green fluorescence of BP and the red fluorescence of mitochondria was observed in the PAHN‐1 group than that in the PAHN‐3 group (**Figure** **3**a). Additionally, the average Pearson's colocalization coefficient (PCC) was calculated to be 0.83 for PAHN‐1 and 0.56 for PAHN‐3 after 6 h of coincubation with 4T1 cells (Figure 3b). To assess cell apoptosis based on mitochondrial targeting, the expression of apoptotic regulators was evaluated by western blotting. It was mentioned that the treatment with both PAHN‐1 + US and PAHN‐3 + US downregulated the expression of the anti‐apoptotic BCL‐2 protein compared with the control, control + US groups, and PAHN‐1 + US upregulated the expression of proapoptotic BAX the most. In particular, mitochondrial damage often triggers the release of cytochrome C (Cyt‐C) from the mitochondria to the cytoplasm, thus activating downstream proapoptotic signals to induce cell death. We found that PAHN‐1 + US induced the highest expression levels of Cyt‐C in cells (Figure 3c). The above results demonstrated that PAHN‐1 could effectively target to mitochondria and thereby induce a more intense apoptotic process than that obtained without mitochondrial targeting capacity. Additionally, we used bio‐TEM to obtain direct insight into the morphological changes in mitochondria. After treatment with PAHN‐1 + US, mitochondria exhibited noticeable chondrosome internal and external cell swelling, whereas they had typical morphological features before treatment (Figure 3d,e). Since mitochondrial membrane potential (MMP) is a vital marker reflecting mitochondrial homeostasis, cells were stained with the mitochondrial probe JC‐1 after different treatments. As revealed in Figure 3f, both CLSM and flow cytometry results showed high and complete red fluorescence, indicating a normal MMP, were observed in the control, control + US and PAHN‐3 groups, while the cells in the PAHN‐3 + US groups emitted moderate green fluorescence, revealing a decreased membrane potential. Furthermore, cells treated with PAHN‐1 + US demonstrated strongly decreased MMP, suggesting highly severe mitochondrial damage. Interestingly, the cells treated by PAHN‐1 without US irradiation similarly demonstrated a relatively low MMP, indicating that the mitochondria in this group were also damaged. This is probably because the KLA sequence can not only target mitochondria, but also disrupt negative charges in the mitochondrial membrane, leading to a decrease in MMP.^[^ ^26^ ^]^ Mitochondria are the primary site of aerobic respiration in cells. Inspired by this, we detected the hypoxic condition of cells after PAHN‐1 treatment. As shown in Figure 3g, both PAHN‐1 and PAHN‐1 + US significantly inhibited the immunofluorescence of hypoxia‐inducible factor (HIF‐1*α*) in cancer cells compared with the control group, verifying that mitochondrial dysfunction helps to alleviate the tumor hypoxia effect. The improvement of hypoxia could provide more O_2_ resources to enhance ROS production.

### 2.4 Amplified ROS Generation by In Situ Self‐Aggregation

**Figure 4:**
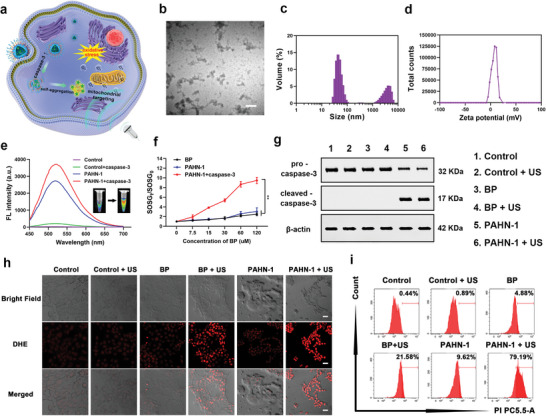
a) Schematic illustration for in situ self‐aggregation and oxidative stress of PAHN‐1 in the presence of caspase‐3. b) TEM images of PAHN‐1 after interaction with caspase‐3. The scale bar is 2 µm. c) Size distribution and d) zeta potentials of PAHN‐1 after interaction with caspase‐3. e) Fluorescence intensity of PBS and PAHN‐1 in the presence and absence of caspase‐3. f) Fluorescence increasing rate of SOSG in BP, PAHN‐1, and PAHN‐1 + caspase‐3 with different concentrations upon US irradiation. g) Intracellular caspase‐3 expression after different treatments analyzed by western blotting. h) CLMS and i) flow cytometry analysis of intracellular ROS generation after different treatments stained by DHE. The scale bars are 25 µm.

It has been demonstrated that achieving AIE, turning from “off” to “on” upon aggregation, by a variety of clever designs is promising in bioapplication. We utilized the caspase‐3‐cleaved sequence DEVD to perform a “stimuli‐responsive self‐aggregation in situ” strategy, which makes BP‐mediated SDT efficacy from “weak” to “strong” (**Figure** **4**a). The HPLC traces revealed that after treatment with human recombinant caspase‐3 protein, the initial peak of PAHN‐1 decreased. A new peak representing hydrophobic substances appeared, indicating that the hydrophilic component was detached from the nanosystem (Figure S10, Supporting Information). TEM imaging revealed that PAHN‐1 exhibited significant aggregation in the presence of caspase‐3, probably because the decrease in water solubility after removing the hydrophilic motif triggers aggregate formation (Figure 4b). Furthermore, the larger size and decreased zeta potential of PAHN‐1 were detected by DLS after caspase‐3 treatment (Figure 4c,d). In addition, we evaluated the fluorescence intensity of PAHN‐1 before and after the addition of caspase‐3. An increased fluorescence value was detected in PAHN‐1 solution after treatment with caspase‐3 for 4 h; however, there was no significant change in PBS solution (Figure 4e). This indicated that a tighter self‐aggregation of PAHN‐1 would enhance the fluorescence signal output. Encouraged by these interesting phenomena, a SOSG probe was used to investigate the ^1^O generation ability of BP and PAHN‐1 in the presence and absence of caspase‐3. We found that as the concentration and time of incubation increased, the growth rate of ^1^O generation in the PAHN‐1 with caspase‐3 group was much greater than that in the BP and PAHN‐1 groups (Figure 4f and Figure S11, Supporting Information). Moreover, as the enzyme and substrate ratio increased, PAHN‐1 generated ^1^O rapidly, while the growth rate of ^1^O in BP group was stable (Figure S12, Supporting Information). These results above suggest that the ingenious introduction of a caspase‐3‐responsive sequence in this nanosystem is capable of triggering self‐aggregation in situ, thus enhancing ROS production via AIE effect. Next, intracellular active caspase‐3 expression was investigated after different treatments. The PAHN‐1 + US group showed an obviously upregulated expression of cleaved caspase‐3 and downregulated expression of procaspase‐3 because of the activation of a cell apoptotic process by SDT. Surprisingly, the PAHN‐1 group also revealed upregulated cleaved caspase‐3 expression (Figure 4g). We speculated that the disturbance of MMP caused by PAHN‐1 simultaneously triggers an increase in the expression level of caspase‐3.^[^ ^27^ ^]^ The high caspase‐3 level in cells ensures the implementation of the “in situ self‐aggregation” strategy. Finally, the intracellular content of ROS was detected by dihydroethidium (DHE) and 2,7‐dichlorodihydrofuorescein diacetate (DCFH‐DA) for fluorescence imaging. As shown in Figure 4h and Figure S13, Supporting Information, cells in the control group with or without US irradiation showed negligible fluorescence signals. In addition, cells treated with BP + US had a higher fluorescence intensity than those with BP and PAHN‐1 without US irradiation, suggesting the exertion of a sonodynamic effect by BP. Comparatively, abundant ROS was generated in a cell treated with PAHN‐1 + US, which exhibited the brightest fluorescence, indicating the most robust ROS production ability by PAHN‐1. This result confirmed the contribution of peptide–AIEgen synergy to the sonodynamic effect of BP. The quantitative flow cytometry results, which further verified the trends of ROS production before and after US irradiation, were in accordance with the CLSM images (Figure 4i).

### 2.5 Cytotoxicity and Antitumor Efficacy of PAHN In Vitro

**Figure 5:**
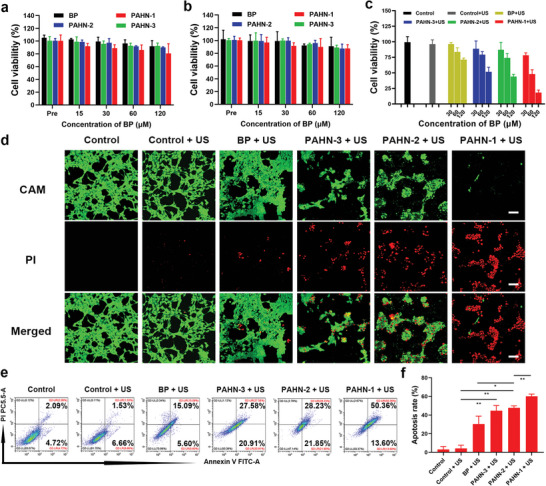
a,b) Cytotoxicity of BP, PAHN‐1, PAHN‐2, and PAHN‐3 against 4T1 cells and HUVECs. c) Relative cell viabilities of 4T1 cells after incubating with different formulations under various concentrations. d) Fluorescent images of 4T1 cells costained with calcein AM and PI after various treatments. The scale bars are 100 µm. e) Apoptosis levels of cells and f) corresponding quantitative analysis evaluated by flow cytometry after various treatments. Data are presented as mean ± SD, *n* = 3.

The biosafety of free BP and three hybrid nanosystems at various concentrations was first evaluated by CCK‐8 assay before cytotoxicity assessment. Without US irradiation, there was no evident change in the survival of 4T1 cells treated with BP, PAHN‐2, and PAHN‐3. PAHN‐1 partially suppressed the growth of tumor cells when the concentration of BP was up to 120 µm, even though the viability of cells was still higher than 80% in this condition (**Figure** **5**a). We speculated that THPP and the mitochondrial targeting sequence in PAHN‐1 promote nanosystem intracellular accumulation and MMP damage, which may have an influence on cell survival. Notably, PAHN‐1 exhibited negligible cytotoxicity against HUVECs and inoblast L929 (Figure 5b and Figure S14, Supporting Information). This can be explained by that normal cells have relatively low nanohybrids uptake compared with tumor cells, and the impact on cell survival is therefore negligible. These results suggested that the rationally designed PAHN has desirable biocompatibility with nontumor tissue or organs. To study the antitumor effect, the viabilities of 4T1 cells after different treatments were evaluated. Treatment with free BP plus US activation showed an unsatisfactory antitumor efficacy, which could be ascribed to poor intracellular internalization and limited ROS generation. Cell viabilities in the PAHN‐2 + US and PAHN‐3 + US groups were relatively lower than those in the BP + US group. Impressively, the cell viability in PAHN‐1 + US group decreased sharply to less than 20%, demonstrating the significantly greater antitumor efficacy than any other treatment tested (Figure 5c). Moreover, to visually evaluate the SDT‐mediated cell apoptosis, we stained the cells with propidium iodide (PI) and calcein AM to determine the dead and live cells. The largest number of dead cells emitting red fluorescence was essential observed in the PAHN‐1 + US group, indicating that this treatment led to maximal cell damage (Figure 5d). Quantitative cell apoptosis analysis performed by flow cytometry showed the same trend. The highest apoptosis ratio of tumor cells occurred in the treatment with PAHN‐1 + US (Figure 5e,f). The significant difference in antitumor efficacy between peptide–AIEgens hybrids and AIE molecules can be attributed to two factors. On the one hand, tumor homing and penetrating sequences and mitochondria targeting sequences realize enhanced targeting capability of the nanosystem from tumor cells to subcellular organelles and induce mitochondria‐related cell death. On the other hand, upregulated caspase‐3 expression further cleaves the enzyme‐responsive sequence, thus initiating self‐aggregation of the nanosystem for ROS amplification.

### 2.6 ICD Initiated by Mitochondria‐Mediated Oxidative Stress

**Figure 6:**
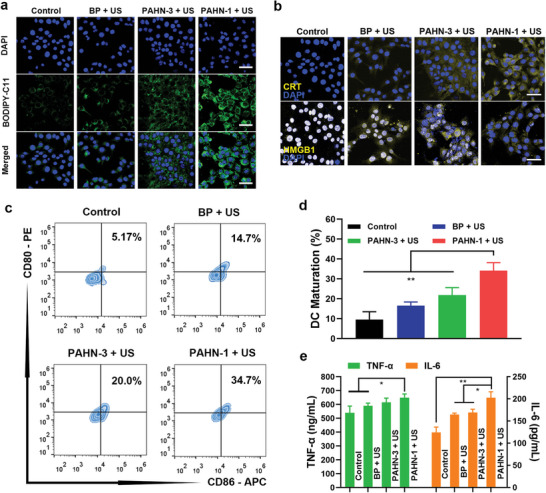
a) Lipid peroxidation level of 4T1 cells stained by BODIPY‐C11 after various treatments. b) Fluorescent images of intracellular CRT and HGMB1 exposure of 4T1 cells after various treatments. c) Flow cytometry and d) corresponding quantitative analysis of DC maturation in different groups. e) The release levels of TNF‐*α* and IL‐6 in cell supernatants after corresponding treatments. Data are presented as mean ± SD, *n* = 3.

The ICD process is capable of reversing the immunogenicity from inactive to active. Although it is well‐established that activating endoplasmic reticulum (ER) stress is important to induce ICD, a previous study has reported that mitochondrial oxidative stress also plays a vital role in ICD induction.^[^ ^28^ ^]^ Moreover, AIEgens that can increase ROS levels by light or sound activation have also been reported as effective ICD inducers. Therefore, we investigated the effect of PAHN stimulation on ICD. The peroxidation of membrane lipids, which is considered as an indicator of oxidative stress, was initially detected by a lipid peroxidation (LPO) sensor, BODIPY‐C11. Compared with the control group, the slight green fluorescence could be observed in the BP + US and PAHN‐3 + US, groups which was attributed to SDT‐mediated cell death. Notably, a stronger green fluorescence was detected in the cells treated with PAHN‐1 + US than in those treated with BP + US and PAHN‐3 + US (**Figure** **6**a). This result suggested that cell death based on mitochondrial damage can lead to amplified oxidative stress. The translocation of calreticulin (CRT) and high mobility group box 1 (HMGB1) is one of most crucial hallmark events during the ICD process. These molecules serve as “eat me” signals to facilitate dendritic cell (DC)  phagocytosis of the corpses and debris of cancer cells for antigen presentation. Similar to the results above, 4T1 cells treated with PAHN‐1 + US had the most apparent CRT exposure on the cell membrane. Moreover, PAHN‐1 + US induced the most significant HMGB1 release from the nucleus to the extracellular environment compared to that in the other groups, indicating the superior performance of PAHN‐1 in triggering the release of damage‐associated molecular patterns (DAMPs) and ICD induction (Figure 6b). To further investigate the maturation potential of DCs activated by the ICD, Transwell systems were used, in which cell debris was seeded in the upper chambers after different treatments and immature DCs (iDCs) were seeded in the lower chambers. The results showed that the percentage of matured DCs could increase from 9.59% (control) to 16.55%, and 21.81% after treatment with BP + US and PAHN‐3 + US, respectively. More importantly, DC maturation in the PAHN‐1 + US group (34.16%) was significantly higher than in the other three groups (Figure 6c,d). The highest levels of secreted interleukin 6 (IL‐6) and tumor necrosis factor alpha (TNF‐*α*) were also detected in the PAHN‐1 + US group, as shown in Figure 6e. These data indicated that the sonodynamic effect originating from PAHN‐1 can provoke vigorous ICD‐related immune responses therefore, should confer an excellent therapeutic effect against tumor invasion and metastasis.

### 2.7 SDT Efficacy and Antitumor Immunity of PAHN In Vivo

**Figure 7:**
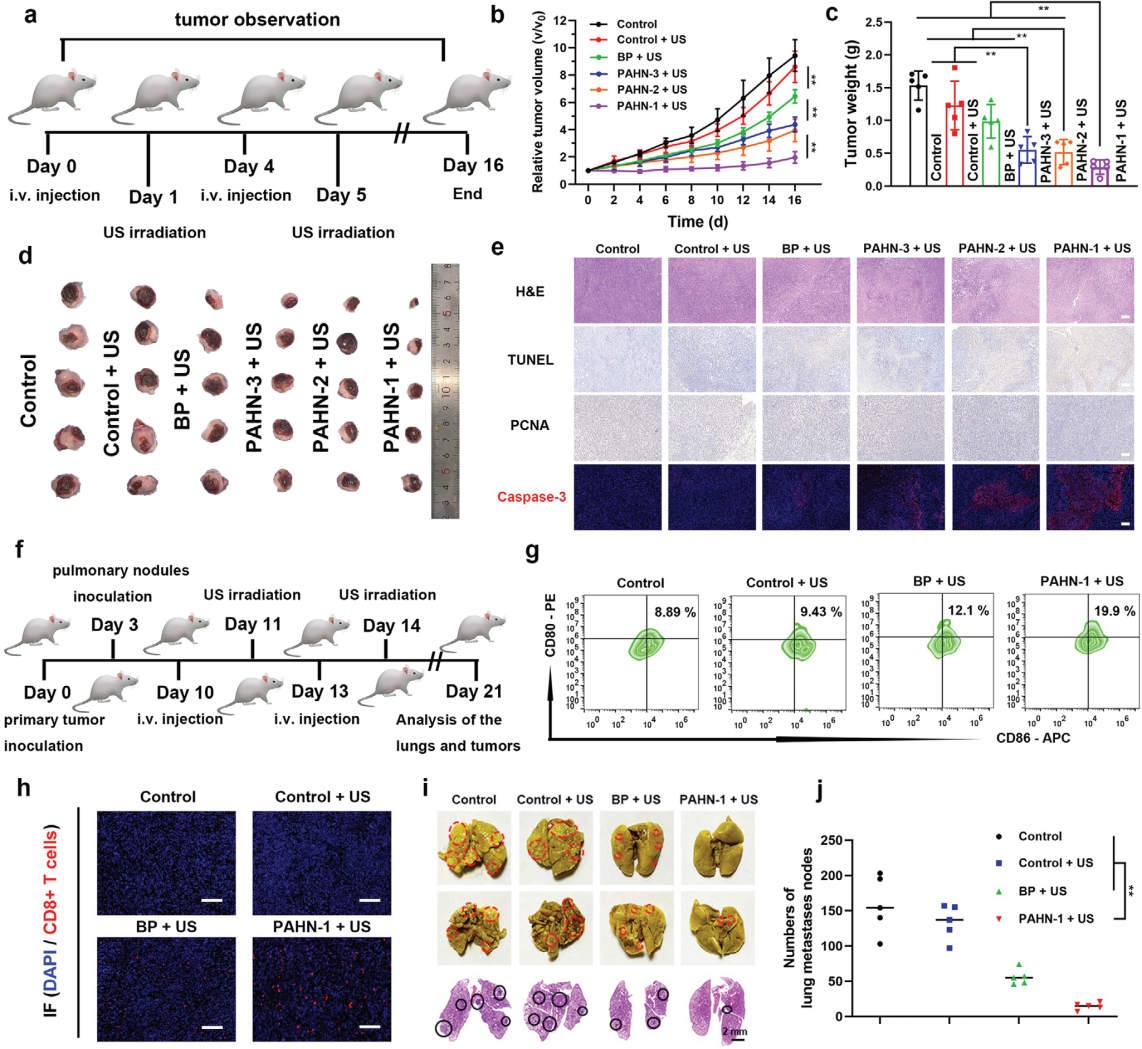
a) Schematic on the administration and US irradiation time for in vivo therapy. b) Relative tumor growth curves and c) tumor weights of 4T1 tumor‐bearing mice after various treatments. d) Digital pictures of 4T1 tumors after receiving different treatments. e) H&E, TUNEL, PCNA, and caspase‐3 staining of tumor sections from various treatment groups. The scale bars are 100 µm. f) Schematic illustration of US‐triggered therapy to initiate an antitumor immune response and suppress lung metastasis. g) Flow cytometry analysis of DC maturation in vivo after different treatments. h) Immunofluorescence images of CD8^+^ (red) T cells in primary tumors after different treatments. The scale bars are 100 µm. i) Gross appearance and H&E staining of lungs and j) the number of pulmonary nodules after receiving different treatments. Data are presented as mean ± SD, *n* = 5.

Encouraged by the effective targeting, ROS enhancement and ICD activation of PAHN‐1 in vitro, we next investigated the performance of antitumor effect in vivo. Female BALB/c mice bearing subcutaneous tumors were randomly divided into six groups (*n* = 5): Group 1, control; Group 2, control + US; Group 3, BP + US; Group 4, PAHN‐3 + US; Group 5, PAHN‐2 + US; and Group 6, PAHN‐1 + US. The tumor size and body weight were recorded every two days (**Figure** **7**a). As shown in Figure 7b,d, both the control and control + US groups showed fast and uncontrollable tumor growth, with 9.42‐fold and 8.60‐fold increases compared with the original tumor volume, in contrast the tumors treated with BP + US, PAHN‐3 + US, and PAHN‐2 + US displayed moderate tumor growth inhibition owing to the sonodynamic effect of BP. As expected, the mice treated with PAHN‐1 + US exhibited a prominent antitumor effect, with only a 1.96‐fold increase in the original tumor volume. In addition, the weights of tumors excised from mice after 16 days of treatment were also consistent with the trend compared to the tumor volume changes (Figure 7c). To further confirm the therapeutic effects of each treatment, the hematoxylin and eosin (H&E), proliferating cell nuclear antigen (PCNA) and TdT‐mediated dUTP nick‐end labeling (TUNEL) staining of tumor sections were also performed. Following a similar trend to that described above, the PAHN‐1 + US treated group exhibited the most severe cell necrosis compared with the other groups. The number of apoptosis‐positive cells that were recorded as dark brown nuclei in the TUNEL assay was also the highest in the PAHN‐1 + US group. In contrast, the proliferation‐positive cells stained by PCNA were the least abundant. We also detected caspase‐3 expression in tumor sections by immunofluorescence staining. The results revealed that the caspase‐3 expression showed the same features observed in vitro; that is, the tumor treated with PAHN‐1 + US had the highest caspase‐3 expression (Figure 7e). These results immediately suggested that PAHN‐1 has superior performance in the inhibition of tumor growth in vivo. In addition, H&E staining revealed no pathological lesions in major organs after different treatments (Figure S15, Supporting Information). The body weight of the mice did not significantly differ during the therapy period (Figure S16, Supporting Information). We have demonstrated that PAHN‐1 + US is capable of stimulating intense ICD process of 4T1 cells in vitro. To further verify whether the immune response is evoked to enhance therapeutic efficacy in vivo, xenografts and lung metastatic models were successively established (Figure 7f). On day 7 after the second treatment, tumor tissues were dissected to prepare a single‐cell suspension for evaluating mature DC levels in vivo. Flow cytometry analysis showed that in contrast to the control group (8.62%), the average percentage of DC maturation was slightly elevated to 9.63% in the BP + US group. Interestingly, mature DCs in the group treated with PAHN‐1 + US significantly increased to 14.53% (Figure 7g and Figure S17, Supporting Information). ELISA revealed that the secretion of cytokines, such as IL‐6, TNF‐*α* and interferon‐gamma (IFN‐*γ*), was the highest in the group of PAHN‐1 + US, suggesting successful initiation of the systemic immunity (Figure S18, Supporting Information). Furthermore, numerous FITC‐stained CD8^+^ T cells that represent the formation of cytotoxic T lymphocytes (CTLs) were observed in tumors treated with PAHN‐1 + US by using an immunofluorescence assay (Figure 7h). We reasoned that mature DCs activated by ICD would present tumor‐associated antigens to activate CD8^+^ cells and thus enhance the adaptive immune responses. Given this fact, gross appearance observation and H&E analysis of lung tissues were conducted to evaluate the antimetastatic effect. As shown in Figure 7i, lungs in the control and control + US groups were occupied by a mass of metastatic nodules, whereas the BP + US and PAHN‐1 + US treatments inhibited lung metastasis to different degrees. The quantitative results confirmed that the PAHN‐1 + US group had the fewest lung metastatic lesions, 14.6 ± 5.2 nodules, compared with the control, 159.0 ± 41.1; control+ US, 133.8 ± 24.8; and BP + US groups, 56.0 ±11.1, suggesting that PAHN‐based SDT promoted a powerful immune effect against tumor metastasis (Figure 7j). Moreover, the survival curve demonstrated that the tumor‐bearing mice treated with PAHN‐1 + US had the longest survival time during the 60‐day monitoring period than the mice in the other three groups, indicating that the long‐term survival rate was improved by PAHN‐1 + US treatment (Figure S19, Supporting Information).

### 2.8 Biosafety Assay of PAHN In Vivo

**Figure 8:**
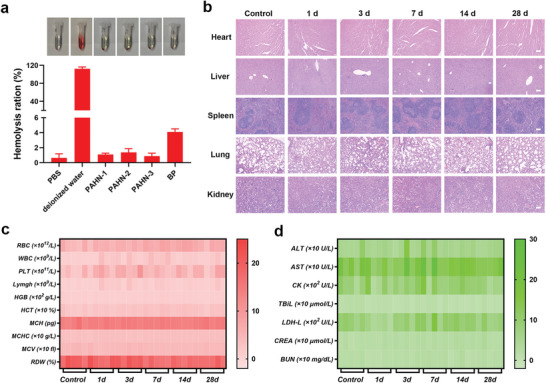
a) Hemolysis evaluation after treating with PBS, deionized water, BP, PAHN‐3, PAHN‐2, and PAHN‐1. b) H&E staining of the major organs in mice sacrificed at different time intervals after intravenous administration of PAHN‐1. The scale bars are 100 µm. Heat map of c) routine blood and d) blood biochemistry analysis in mice sacrificed at different time intervals after intravenous administration of PAHN‐1. Data are presented as mean ± SD, *n* = 5.

Finally, in vivo biosafety of PAHN was systematically evaluated. Blood hemolysis tests revealed that compared to the relatively high hemolysis (4.1%) induced by free BP, the hemolysis ratios of all nanosystems were less than 1.5%, revealing improved biocompatibility (**Figure** **8**a). There were neither significant histopathological changes nor abnormal inflammatory responses in the major organs after intravenous injection of PAHN‐1 in both the short and long term (Figure 8b). Moreover, the hematological indices showed negligible variations at the tested dose compared to the control group, which offers promise in clinical translation (Figure 8c,d).

## 3 Conclusion

In summary, this study reports a suitably constructed PAHN aimed at inhibiting the tumor invasion and metastasis of breast cancer. Stratified targeting and in situ self‐aggregation were concurrently achieved to strengthen AIEgen‐involved SDT. After intravenous injection, PAHN‐1 preferentially accumulated and penetrated through the tumor stroma barrier, mediated by the THPP sequence. Then, the mitochondrial targeting motif drove PAHN‐1 into the mitochondria after cell internalization, leading to upregulated active caspase‐3 expression via mitochondrial dysfunction. More importantly, PAHN‐1 exhibited in situ self‐aggregation after cleavage by active caspase‐3 and enabled BP to generate more ROS in a more tightly aggregated state, enhancing SDT. Moreover, due to the imbalance of ROS in the mitochondria, PAHN‐1‐mediated SDT induces a severe oxidative stress reaction, which can initiate a more extensive ICD process than that mediated by BP molecules and moreover evokes a powerful antitumor immune response to simultaneously suppress tumor proliferation and metastasis. Profiting from the intrinsic biocompatibility and biofunction‐editing properties of peptide‐based supramolecular assemblies, PAHN provides a prospective idea for augmenting AIEgen‐related antitumor therapeutics.

## 4 Experimental Section

### Reagents and Materials

The hydrophobic bis‐pyrene unit (BP‐COOH) was synthesized by Apeptide Biotech Co., Ltd (shanghai, China) according to a previous report.^[^ ^29^ ^]^ Murine mammary carcinoma (4T1), HUVECs, and L929 cell lines were purchased from the MEIXUAN Biological science and technology Co., Ltd. Nile red (NR) was purchased from Shanghai Macklin Biochemical Co., Ltd. (China). Recombinant human caspase‐3 protein was purchased from Sino Biological, Inc. (China). BODIPY‐581/591C11 probe was purchased from Thermo Fisher Scientific Co., Ltd (Carlsbad, California, U.K.). Propidium iodide(PI), calcein AM, and Cell Counting Kit‐8 were purchased from Dojindo (Tokey, Japan). FITC‐CD11c^+^, PE‐CD80^+^, and APC‐CD86^+^ antibodies were purchased from Biolegend, (California, MA). TNF‐*α*, IFN‐*γ*, and IL‐6 ELISA kits were purchased from Uscn Life Science (China).

### Synthesis of PAHN‐1, 2, and 3

In brief, CFFFVLKLAKLAKDEVDAKRGARSTA‐Beads, CFFFVLKKKDEVDAKRGARSTA‐Beads, and CFFFVLKLAKLAKDEVDAKDGAESHH‐Beads were first synthesized by standard solid‐phase peptide synthesis (SPPS) techniques. Then amino protection group (Dde) was removed for 5 h using hydrazine hydrate (2%, v/v)/DMF solution. BP‐COOH was conjugated to the main chain of peptide 1–3 by amide condensation reaction. Next, peptides were cut off from the beads for 2 h using the solution containing trifluoroacetic acid (TFA, 95%, v/v), triisopropylsilane (TIPS, 2%, v/v), 1,2‐ethanedithiol (EDT, 2%,v/v), and H_2_O (1%, v/v). After cold ether precipitation, the dry peptides were freeze‐dried and then purified by HPLC (Waters, ZQ2000, China). The synthesis of end products was determined by mass spectrometer (MS, BAIIENS, AB5800, China) and nuclear magnetic resonance spectroscopy (NMR, AVANCE III, Bruker, Germany).

### Preparation and Characterization of PAHN‐1, 2, and 3

PAHN‐1, 2, and 3 were dissolved in different volumes (10, 20, 100, 200, 400, 600, 800, 900, 980, and 990 µL) of DMSO and then quickly mixed with deionized water, making the final volume of mixture reach 1 mL. The ultraviolet and fluorescence spectrum of each sample were determined by a multifunctional microplate reader (M3, SpectraMax, China). Next, the nanosystem containing 1% DMSO and 99% water solution were used in the following experiments. The morphologies of PAHN‐1, 2, and 3 were observed by using TEM (Hitachi H‐7600, Japan). Zeta potentials and size distribution were determined by a DLS (Malvern, NanoZS, UK).

### CAC Determination

NR molecule was employed to determine the CAC of nanosystem. First, 50 µL of NR solution in acetone (1 × 10^−4^ m) was added to a 5 mL centrifuge tube. The tube was kept overnight to evaporate the acetone completely, followed by the addition of a series of concentrations of PAHN‐1, 2, and 3 (0.03, 0.06, 0.12, 0.3, 0.6, 1.2, 3, 12, and 30 µm). The concentration of NR was fixed at 1 × 10^−6^ m at last. After mixing NR with PAHN solution and maintaining at 37 °C for 12 h. The fluorescence intensity of NR (Ex/Em = 550 nm/620 nm) in different mixtures were recorded and plotted to determine the CAC values.

### Cell Culture and Tumor‑Bearing Mice Model Establishment

The murine 4T1 breast cancer cell and human umbilical vein endothelial cell (HUVEC) were cultured using RPMI‐1640 medium supplemented with 10% fetal bovine serum, 100 U·mL^−1^ penicillin, and streptomycin under 5% CO_2_ atmosphere at 37 °C. All female balb/c mice (16 to 20 g, 4 to 6 weeks) were purchased from Ensiweier Biotechnology Co., Ltd (Chongqing, China) and bred in humid conditions. Food and water were free to provide. All the animal experimental procedures were in accordance with the ethical standards of Chongqing Medical University. 4T1 tumor‐bearing mice were established by subcutaneously injecting PBS solution (100 µL), suspended with 1 × 10^6^ 4T1 cells, into the right mammary fat pad. The tumor volumes were calculated as 0.5 × length × width^2^.

### Tumor Targeting and Tumor Spheroid Penetrating Efficiency

4T1 cells were seeded into a laser confocal cell‐culture dish at a density of 1 × 10^5^ cells and incubated for 24 h. Then the cells were incubated with the medium containing either 60 µm of BP, PAHN‐2 or PAHN‐1 for 0.5, 2 and 4 h, respectively. To observe cellular uptake behavior, the cells were fixed in 4% formaldehyde for 15 min and stained with DAPI for 10 min. Finally, CLSM was performed to observe the fluorescent images. For the quantitative analysis of intracellular uptake, 4T1 cells were seeded into 12‐well plates at a density of 1 × 10^5^ cells for 24 h incubation. After that, the culture medium was replaced by a fresh medium containing BP, PAHN‐2 and PAHN‐1 for another 0.5, 2 and 4 h incubation, respectively. Cells were collected and resuspended in PBS for quantitative determination via flow cytometry (Becton Dickinson, FACS Vantage, USA). To establish 3D spheroids, 4 × 10^6^ 4T1 cells were seeded in 6‐well ultra‐low adherent plates for 7 days of formation. The spheroids were further cultured in a medium containing 60 µm of either BP, PAHN‐2, or PAHN‐1 for 4 h. Penetration depth was measured by multiple level scans under CLSM.

### Detection of Mitochondrial Colocalization and Depolarization

Cells were seeded into a laser confocal culture dish at a density of 1 × 10^5^ cells. Then cells were incubated with the medium containing 60 µm of PAHN‐3 and PAHN‐1 for 4 h, respectively. After that, cells were fixed for 15 min with 4% formaldehyde, and stained by DAPI and mitochondria‐tracker Red (Beyotime Biotechnology) for 30 min. The colocalization analysis was performed using ImageJ software. JC‐1 dye was used to investigate MMP. First, 4T1 cells were seeded in cell‐culture dishes at a density of 1 × 10^5^, followed by 4 h incubation with either PBS, PAHN‐3 (60 µm) or PAHN‐1 (60 µm). Then cells were exposed to US irradiation at an intensity of 3 W cm^−2^ and a frequency of 1 MHz for 4 min. After 4 h another incubation was done, the medium was replaced by a fresh medium containing JC‐1 staining solution and incubated for 20 min. The variations in the MMP were detected by using CLMS and flow cytometry.

### Western Blotting Assay

To investigate mitochondria‐related cell apoptosis, the expression of Cyt‐C, BAX, and BCL‐2 protein were detected by western blotting analysis. Cells were coincubated with a culture medium containing PBS, PAHN‐3 (60 µm) or PAHN‐1 (60 µm) for 4 h, followed by US irradiation. After another 6 h incubation, the cells were harvested and lysed with RIPA buffer containing a protease inhibitor cocktail. The cell lysates were collected at 10 000 rpm for 10 min and the protein concentrations were adjusted with a BCA Protein Assay Kit. Quantified protein lysates were resolved on sodium dodecyl sulfate‐polyacrylamide gel electrophoresis (SDS‐PAGE) gel, and transferred onto the PVDF membrane. The membranes were blocked with 5% nonfat dry milk for 2 h and then immunoblotted with primary antibodies including Cyt‐C, BAX, and BCL‐2 overnight at 4 °C. After three washes using buffered saline with 0.05% Tween‐20, the membranes were subsequently incubated with a secondary antibody (dilution 1:2000) for 1 h at room temperature. The immune blots were visualized using an enhanced chemiluminescence western blotting detection kit.

### ESR Measurement

Briefly, DMPO (50 mm, Sigma‐Aldrich) was added to the solution with BP (120 µm) or PAHN‐1 (120 µm). The mixtures treated with or without US irradiation (3 W cm^−2^, 4 min) were transferred to a quartz tube. Then, the ESR signal was obtained on a Bruker A300‐10/12 spectrometer (Bruker, Billerica, MA). The ESR signal of PBS treated with US irradiation was also detected as a control.

### Generation of Singlet Oxygen Enhanced by Caspase‐3

Generation of singlet oxygen (^1^O_2_) was determined using a SOSG probe. First, 1 µL of SOSG dissolved in methanol (5 mm) was in time mixed with 2 mL of various concentrations (0, 7.5, 15, 30, 60, and 120 µm) of BP or PAHN‐1 or PAHN‐1 with recombinant human caspase‐3 proteins (125 ng mL^−1^). The fluorescence intensities (Ex/Em = 488 nm/525 nm) of mixtures that received US treatment (3 W cm^−2^, 4 min) were monitored by microplate reader. ROS production under different US irradiation time and caspase‐3 content were also investigated. Briefly, the SOSG solution was added into the 30 µm of BP or PAHN‐1 with different content of caspase‐3 (enzyme substrate ratio: 0, 0.25, 0.5, 1, 2, and 4), and then the mixture was irradiated by US for different time (0, 30, 60, 120, 240, and 300 s).

### Intracellular ROS Detection

The cells seeded in confocal culture dish or microplates were randomly distributed into six groups, which were then coincubated with PBS, BP (60 µm), and PAHN‐1 (60 µm) for 4 h. The cells in control, BP + US, and PAHN‐1 + US groups were then exposed to US irradiation at 3 W cm^−2^ for 4 min. After another 4 h incubation, fluorescent dye DHE and DCFH‐DA were used to detect ROS levels in tumor cells. All dishes were imaged by CLSM, and cells were collected for flow cytometry analysis.

### In Vivo and Ex Vivo Fluorescence Imaging

For the fluorescence imaging and biodistribution assessment in vivo, DIR‐labelled nanosystem was prepared. Briefly, 2 µL of DIR stock solution (2 mm) was added to 1 mL of the PAHN. After stirring for 12 h, the DIR‐labelled nanosystem was finally obtained after dialyzing against deionized water using a dialysis bag (MWCO: 3500 Da) overnight to remove excess DIR. 4T1 tumor‐bearing mice (*n* = 3) were intravenously injected with DIR‐labelled (Ex/Em = 748 nm/780 nm) PAHN‐1 or PAHN‐2 at a dose of 2.4 µmol kg^−1^. Fluorescence images were acquired at preinjection, and 0, 0.5, 1, 2, 6, 24 and 48 h postinjection by using a fluorescence imaging system (CRi Inc., USA). The tumor tissues and major organs were isolated and obtained for ex vivo imaging on the day of animal sacrifice. The corresponding fluorescence intensities were recorded.

### Antitumor Effect In Vitro

To determine the antitumor efficacy in vitro, the 4T1 cells were divided into the following 6 groups: i) control, ii) control + US, iii) BP + US, iv) PAHN‐3 + US, v) PAHN‐2 + US, and vi) PAHN‐1 + US. Then, the medium were replaced with the fresh medium containing corresponding formulations at different concentrations (30, 60, 120 µm). After 4 h of treatment, the cells were rinsed with PBS and then treated by US at an intensity of 3 W cm^−2^ for 4 min. After that, the culture medium was replaced by a fresh serum‐free medium for another 4 h incubation. Finally, a standard CCK‐8 assay was used to evaluate the viability of the cells. The therapeutic effect was also detected by cellular apoptosis assay. In brief, the 4T1 cells were divided into the 6 groups as described above. Next, cells were treated with either BP, PAHN‐3, PAHN‐2, PAHN‐1 at a concentration of 120 µm, or PBS, followed by US irradiation. Finally, these cells were costained by a dye solution of calcein AM (2 µm) and PI (2 µm) or collected for apoptosis analysis using flow cytometry.

### Detection for Lipid Peroxidation and Translocation of CRT

4T1 cells were seeded into a laser confocal culture at a density of 1 × 10^5^ dish and incubated for 24 h. Then the cells were incubated with the medium containing either 60 µm of PAHN‐1, PAHN‐3, or BP for 4 h. The cells in BP + US, PAHN‐3 + US, and PAHN‐1 + US groups were then exposed to US irradiation at 3 W cm^−2^ for 4 min. After another 4 h incubation, cells were stained with of BODIPY‐C11 (2 µm) for 10 min. Then, cells were fixed with 4% paraformaldehyde and blocked by goat serum for 30 min. Afterward, cells were incubated with the primary antibody (Calreticulin Rabbit pAb [A1066], abclonal, China) at 4 °C overnight and incubated with the Dylight‐649‐labelled secondary antibodies at 37 °C for 1 h. Nuclei were stained with DAPI for CLSM observation.

### Immunofluorescence Staining for HMGB1 Detection

For immunofluorescence staining, 4T1 cells were seeded into a laser confocal culture dish overnight. After treating with either PBS, BP, PAHN‐3 or PAHN‐1 (60 µm) followed by US irradiation, cells were fixed with 4% paraformaldehyde for 15 min. Then, cells were permeabilized with 0.5% Triton X‐100 for 10 min and blocked by goat serum for 30 min. Afterward, cells were incubated with the primary antibody (HMGB‐1 Rabbit mAb [A19529], abclonal, China) at 4 °C overnight and incubated with the Dylight‐649‐labelled secondary antibodies at 37 °C for 1 h. Nuclei were stained with DAPI for CLSM observation.

### DC Maturation Experiment In Vitro

The transwell system was used to coculture iDCs and 4T1 cells. Typically, the iDCs were seeded in the lower layer. 4T1 cells receiving different treatments were seeded in the upper layer of the system and cocultured with iDCs for 24 h. Then, iDCs were collected and stained with FITC‐CD11c PE‐CD80^+^ and APC‐CD86^+^ for flow cytometry analysis. The supernatant was collected for ELISA assay analysis. The specific groups were as follows: i) control, ii) BP + US, iii) PAHN‐3 + US, and iv) PAHN‐1 + US

### Tumor Growth and Metastasis Inhibition In Vivo

To evaluate the antitumor efficacy in vivo, thirty 4T1 tumor‐bearing mice were randomly divided into six groups (*n* = 5): i) control, ii) control + US, iii) BP + US, iv) PAHN‐3 + US, v) PAHN‐2 + US, and vi) PAHN‐1 + US. The corresponding agents were administered to tumor‐bearing mice at an equivalent dose of 2.4 µmol kg^−1^ per mouse via tail vein. Twenty four hours later, the regions of the tumors were irradiated by US (3 W cm^−2^, 1 MHz) for 5 min. Mice in the control group were only administered with saline solution (200 µL). The treatment was repeated on day 4. Tumor volumes and body weights were recorded every other day during a 16‐day observation window. The targeted tumor tissues and major organs and were dissected for H&E staining after the treatment ended. Tumor tissue was further stained with caspase‐3, PCNA, and TUNEL. To investigate the anti‐metastasis effect evoked by ICD, xenografts and lung metastasis models were successively established. At first, 4T1 cells (1 × 10^6^ cells per mouse) suspended in PBS solution (100 µL) were subcutaneously inoculated under the right mammary fat pads of twenty BALB/c mice as the primary tumor. Three days later, 4T1 cells (1 × 10^6^) were administered to each mouse via tail vein. These mice were randomly divided into four groups : i) control, ii) control + US, iii) BP + US, and iv) PAHN‐1 + US. Then, the treatments were followed as same as the aforementioned procedures. At day 11 of post‐treatment, lungs were harvested and fixed in Bouin's solution. Metastatic nodules in the lungs were counted and analyzed by H&E staining.

### Biosafety Assay of PAHN In Vivo

To investigate the short and long‐term biosafety of PAHN‐1 in vivo, PAHN‐1 at a dose of 2.4 µmol kg^−1^ was intravenously injected into twenty five healthy mice that were sacrificed at predetermined time points (1, 3, 7, 14, and 28 d postinjection). Five healthy mice that were administrated with the saline solution served as the control group. The blood samples were then collected for biochemistry and routine blood examination. The main organs were stained with H&E.

### Statistical Analysis

Data were expressed as means ± standard deviation. All the statistical analyses were conducted using the SPSS software (version 18.0; IBM Corp., Armonk, NY, USA). Unpaired Student's *t*‐test and Mann–Whitney U test were used to compare significance of differences among groups (*p < 0.05, **p < 0.01).

## Conflict of Interest

The authors declare no conflict of interest.

## Author Contributions

W.J.: Conceptualization, Methodology, Validation, Investigation, Resources, Data curation, Writing original draft: C.C. and X.Q.: Data curation, Visualization, Investigation. L.C. and X.G.: Resources, Investigation. Y.L.: Methodology. J.X.W.: Investigation. J.R.W. and Z.X.: Data curation, Investigation. P.L.: Validation. Z.W. and H.R.: Supervision, Funding acquisition. Z.Z.: Project administration. J.R.: Writing‐Reviewing and Editing, Supervision, Funding acquisition.

## Supporting information

Supporting Information

## Data Availability

The data that support the findings of this study are available from the corresponding author upon reasonable request.

## References

[1] a) M. Li , Y. Gao , Y. Yuan , Y. Wu , Z. Song , B. Z. Tang , B. Liu , Q. C. Zheng , ACS Nano 2017, 11, 3922; 28383899 10.1021/acsnano.7b00312

[2] C. Chen , H. Ou , R. Liu , D. Ding , Adv. Mater. 2020, 32, 1806331. 10.1002/adma.201806331 30924971

[3] Q. Li , Y. Li , T. Min , J. Gong , L. Du , D. L. Phillips , J. Liu , J. W. Y. Lam , H. H. Y. Sung , I. D. Williams , R. T. K. Kwok , C. L. Ho , K. Li , J. Wang , B. Z. Tang , Angew. Chem., Int. Ed. Engl. 2020, 59, 9470. 31557385 10.1002/anie.201909706

[4] a) S. Wang , H. Chen , J. Liu , C. Chen , B. Liu , Adv. Funct. Mater. 2020, 30, 2002546;

[5] Z. Zhang , W. Xu , P. Xiao , M. Kang , D. Yan , H. Wen , N. Song , D. Wang , B. Tang , ACS Nano 2021, 15, 10689. 34077187 10.1021/acsnano.1c03700

[6] a) C. W. Leung , Y. Hong , S. Chen , E. Zhao , J. , J. W. Lam , B. Z. Tang , J. Am. Chem. Soc. 2013, 1, 62; 10.1021/ja310324q 23244346

[7] a) D. Yang , X. Lv , L. Xue , N. Yang , Y. Hu , L. Weng , N. Fu , L. Wang , X. Dong , Chem. Commun. 2019, 55, 15145; 10.1039/c9cc08463k 31790115

[8] X. Qian , Y. Zheng , Y. Chen , Adv. Mater. 2016, 37, 8097. 10.1002/adma.201602012 27384408

[9] L. Zhu , H. Zhou , Z. Zhou , Y. Xia , Z. Wang , H. Ran , P. Li , J. Ren , Nano Lett. 2018, 18, 1831. 29419305 10.1021/acs.nanolett.7b05087

[10] H. W. An , L. L. Li , Y. Wang , Z. Wang , D. Hou , Y. X. Lin , S. L. Qiao , M. D. Wang , C. Yang , Y. Cong , Y. Ma , X. X. Zhao , Q. Cai , W. T. Chen , C. Q. Lu , W. Xu , H. Wang , Y. Zhao , Nat. Commun. 2019, 10, 4861. 31649241 10.1038/s41467-019-12848-5 PMC6813295

[11] a) J. Wang , L. Hu , H. Zhang , Y. Fang , T. Wang , H. Wang , Adv. Mater. 2022, 34, 2104704; 10.1002/adma.202104704 34632634

[12] a) M. T. Jeena , L. Palanikumar , E. M. Go , I. Kim , M. G. Kang , S. Lee , S. Park , H. Choi , C. Kim , Nat. Commun. 2017, 8, 26; 28638095 10.1038/s41467-017-00047-z PMC5479829

[13] a) H. Hunt , L. Simón‐Gracia , A. Tobi , V. R. Kotamraju , S. Sharma , M. Nigul , K. N. Sugahara , E. Ruoslahti , T. Teesalu , J Control Release 2017, 260, 142; 28603028 10.1016/j.jconrel.2017.06.005 PMC6129970

[14] S. Sharma , V. R. Kotamraju , T. Mölder , A. Tobi , T. Teesalu , E. Ruoslahti , Nano Lett. 2017, 17, 1356. 28178415 10.1021/acs.nanolett.6b03815 PMC5819594

[15] a) J. Zhang , Y. Mu , M. Xu , M. F. Foda , H. Han , Chem. Eng. J. 2022, 427, 130775;

[16] a) M. P. Murphy , Trends Biotechnol. 1997, 15, 326; 9263481 10.1016/S0167-7799(97)01068-8

[17] a) P. P. He , X. D. Li , L. Wang , H. Wang , Acc. Chem. Res. 2019, 52, 367; 30653298 10.1021/acs.accounts.8b00398

[18] a) L. Zhang , Y. Wu , X. Yin , Z. Zhu , T. Rojalin , W. Xiao , D. Zhang , Y. Huang , L. Li , C. M. Baehr , X. Yu , Y. Ajena , Y. Li , ACS Nano 2021, 15, 468; 33332957 10.1021/acsnano.0c05065

[19] R. J. Giordano , J. Lahdenranta , L. Zhen , U. Chukwueke , I. Petrache , R. R. Langley , I. J. Fidler , R. Pasqualini , R. M. Tuder , W. Arap , J. Biol. Chem. 2008, 283, 29447. 18718906 10.1074/jbc.M804595200 PMC2570855

[20] a) H. Shi , R. T. Kwok , J. Liu , B. Xing , B. Z. Tang , B. Liu , J. Am. Chem. Soc. 2012, 134, 17972; 23043485 10.1021/ja3064588

[21] a) L. Galluzzi , A. Buqué , O. Kepp , L. Zitvogel , G. Kroemer , Nat. Rev. Immunol. 2017, 17, 97; 27748397 10.1038/nri.2016.107

[22] a) D. V. Krysko , A. D. Garg , A. Kaczmarek , O. Krysko , P. Agostinis , P. Vandenabeele , Nat. Rev. Cancer 2012, 12, 860; 23151605 10.1038/nrc3380

[23] Y. Shin , K. A. Winans , B. J. Backes , S. Kent , J. A. Ellman , C. R. Bertozzi , J. Am. Chem. Soc. 1999, 121, 11684.

[24] W. Jiang , L. Su , M. Ao , X. Guo , C. Cheng , Y. Luo , Z. Xie , X. Wang , J. Wang , S. Liu , Y. Cao , P. Li , Z. Wang , H. Ran , Z. Zhou , J. Ren , J. Nanobiotechnol. 2021, 19, 200. 10.1186/s12951-021-00947-9 PMC8256488 34225744

[25] a) Z. Zhang , W. Xu , P. Xiao , M. Kang , D. Yan , H. Wen , N. Song , D. Wang , ACS Nano 2021, 15, 10689; 34077187 10.1021/acsnano.1c03700

[26] B. Law , L. Quinti , Y. Choi , R. Weissleder , C. H. Tung , Mol. Cancer Ther. 2006, 5, 1944. 16928814 10.1158/1535-7163.MCT-05-0509

[27] M. Sun , C. Wang , M. Lv , Z. Fan , JJ. Du , J. Am. Chem. Soc. 2022, 144, 7337. 35357824 10.1021/jacs.2c00697

[28] C. Chen , X. Ni , S. Jia , Y. Liang , X. Wu , D. Kong , D. Ding , Adv. Mater. 2019, 31, 1904914. 10.1002/adma.201904914 31696981

[29] S. L. Qiao , Y. Wang , Y. X. Lin , H. W. An , Y. Ma , L. L. Li , L. Wang , H. Wang , ACS Appl. Mater. Interfaces 2016, 8, 17016. 27348260 10.1021/acsami.6b04580

