# Supplementary material for: Peptide Supramolecular Assembly‐Instructed In Situ Self‐Aggregation for Stratified Targeting Sonodynamic Therapy Enhancement of AIE Luminogens
Source: Adv Sci (Weinh). 2022 Dec 9;10(4):2204989. doi: 10.1002/advs.202204989 (PMC9896067; doi:10.1002/advs.202204989)
Supplement: Supplementary file 1 — Supporting Information [file ADVS-10-2204989-s001.pdf]

ADVANCED SCIENCE
Open Access

Supporting Information

for *Adv. Sci.*, DOI 10.1002/advs.202204989

Peptide Supramolecular Assembly-Instructed In Situ Self-Aggregation for Stratified Targeting Sonodynamic Therapy Enhancement of AIE Luminogens

*Weixi Jiang, Chen Cheng, Xiaoling Qiu, Li Chen, Xun Guo, Yuanli Luo, Jingxue Wang, Junrui Wang, Zhuoyan Xie, Pan Li, Zhigang Wang, Haitao Ran, Zhiyi Zhou and Jianli Ren**

Peptide Supramolecular Assembly-Instructed In Situ Self-Aggregation for Stratified Targeting Sonodynamic Therapy Enhancement of AIE Luminogens

Weixi Jiang, Chen Cheng, Xiaoling Qiu, Li Chen, Xun Guo, Yuanli Luo, Jingxue Wang, Junrui Wang, Zhuoyan Xie, Pan Li, Zhigang Wang, Haitao Ran, Zhiyi Zhou, and Jianli Ren*

W. Jiang, C. Cheng, X. Qiu, X. Guo, Y. Luo, J. Wang, P. Li, Z. Wang, H. Ran, J. Ren

Department of Ultrasound and Chongqing Key Laboratory of Ultrasound Molecular Imaging, the Second Affiliated Hospital of Chongqing Medical University

No.74 Linjiang Rd, Yuzhong District, Chongqing 400010, P. R. China

E-mail address: renjianli@cqmu.edu.cn (Jianli Ren)

C. Cheng

Department of Ultrasound, Bishan Hospital of Chongqing, Bishan Hospital of Chongqing Medical University

No. 9 Shuangxing Avenue, Biquan Street, Bishan District, Chongqing 402760, P. R. China.

X. Qiu, L Chen

Department of Intensive Care Unit, the Second Affiliated Hospital of Chongqing Medical University

No.74 Linjiang Rd, Yuzhong District, Chongqing 400010, P. R. China

J. Wang

Department of Radiology, the Second Affiliated Hospital of Chongqing Medical University

No.74 Linjiang Rd, Yuzhong District, Chongqing 400010, P. R. China

Z, Xie

Department of Ultrasound, Chongqing General Hospital,

NO. 118 Xingguang Avenue, Liangjiang New Area, Chongqing 401147, P. R. China

Z, Zhou

Department of General practice, Chongqing General Hospital

NO. 118 Xingguang Avenue, Liangjiang New Area, Chongqing 401147, P. R. China

* Corresponding author.

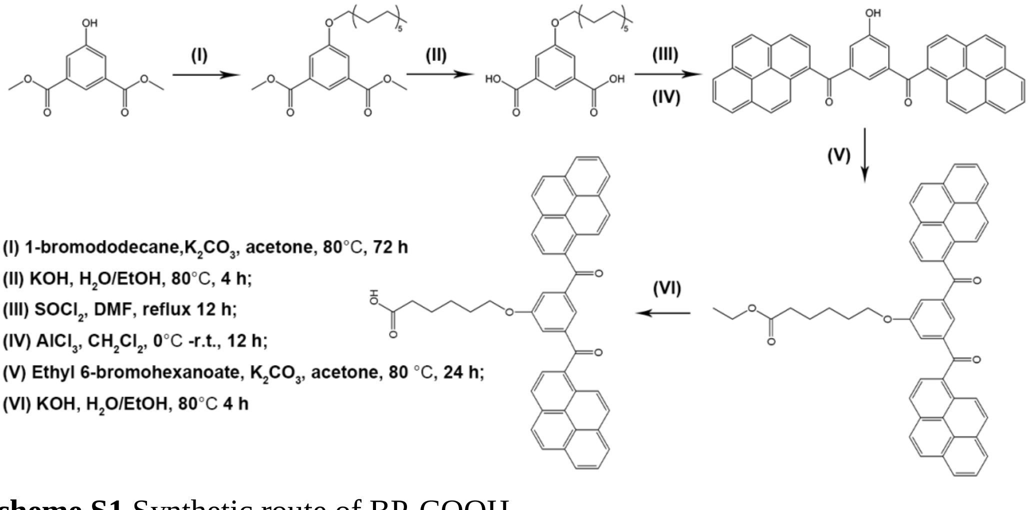

Scheme S1 Synthetic route of BP-COOH.

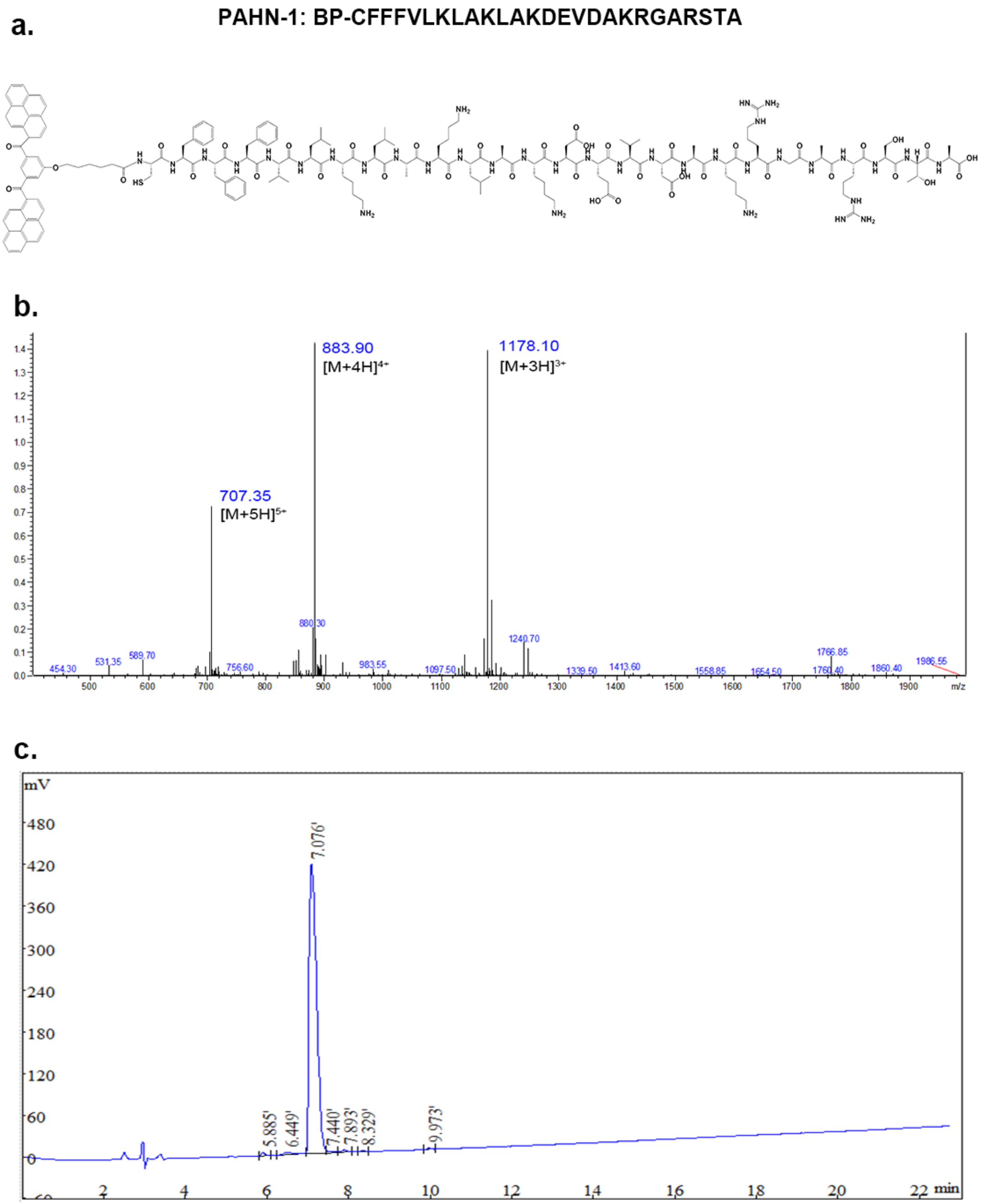

Figure S1. a) The chemical structure, b) mass spectrum and c) HPLC trace of **PAHN-1**. The method of HPLC spectra was as follows: solvent A, 0.1% trifluoroacetic acid in 100% acetonitrile; solvent B, 0.1% trifluoroacetic acid in 100% water; 0.01 min, 36% solvent B, 25 min, 11% solvent B.

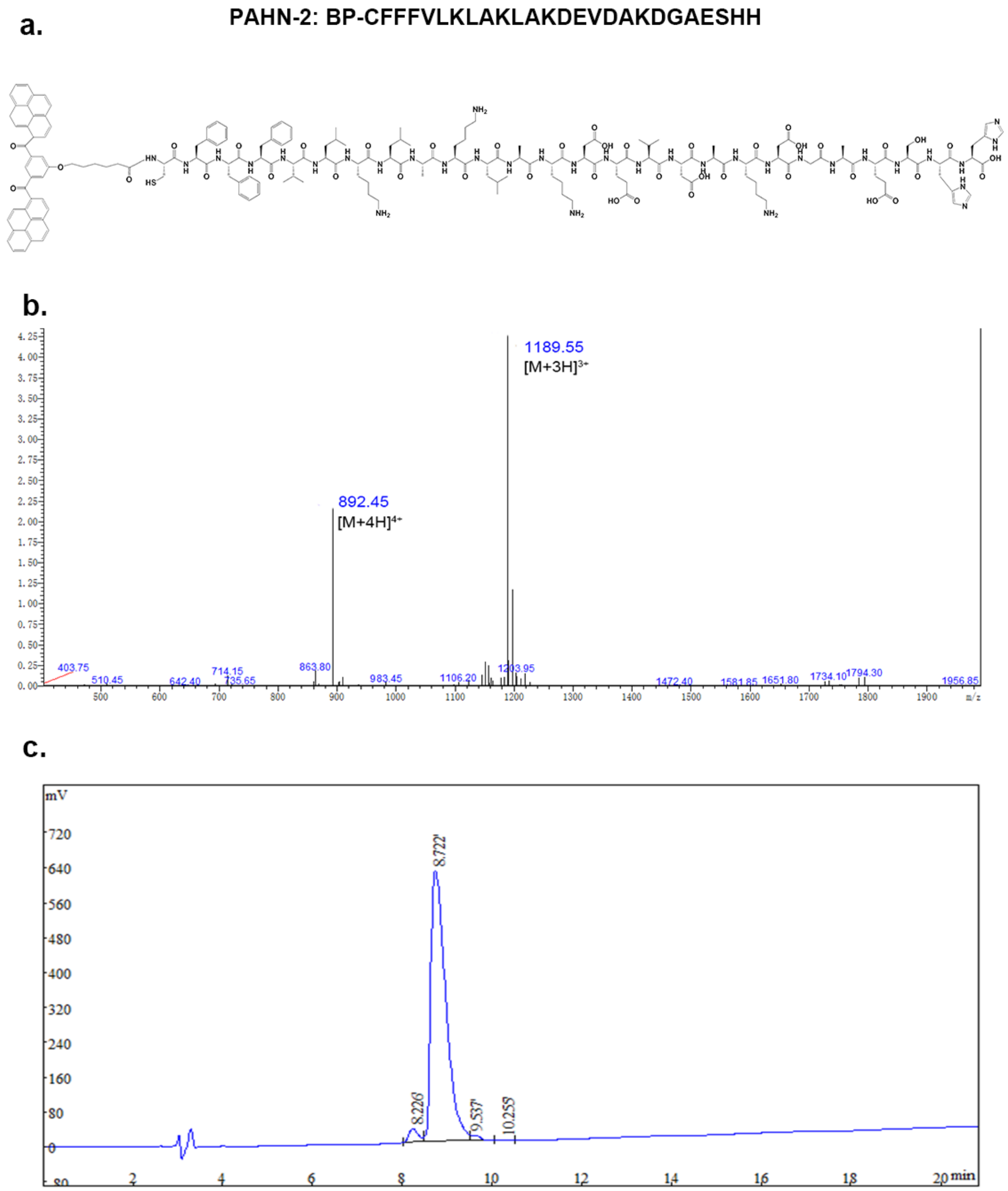

Figure S2. a) The chemical structure, b) mass spectrum and c) HPLC trace of **PAHN-2**. The method of HPLC spectra was as follows: solvent A, 0.1% trifluoroacetic acid in 100% acetonitrile; solvent B, 0.1% trifluoroacetic acid in 100% water; 0.01 min, 85% solvent B, 25 min, 47% solvent B.

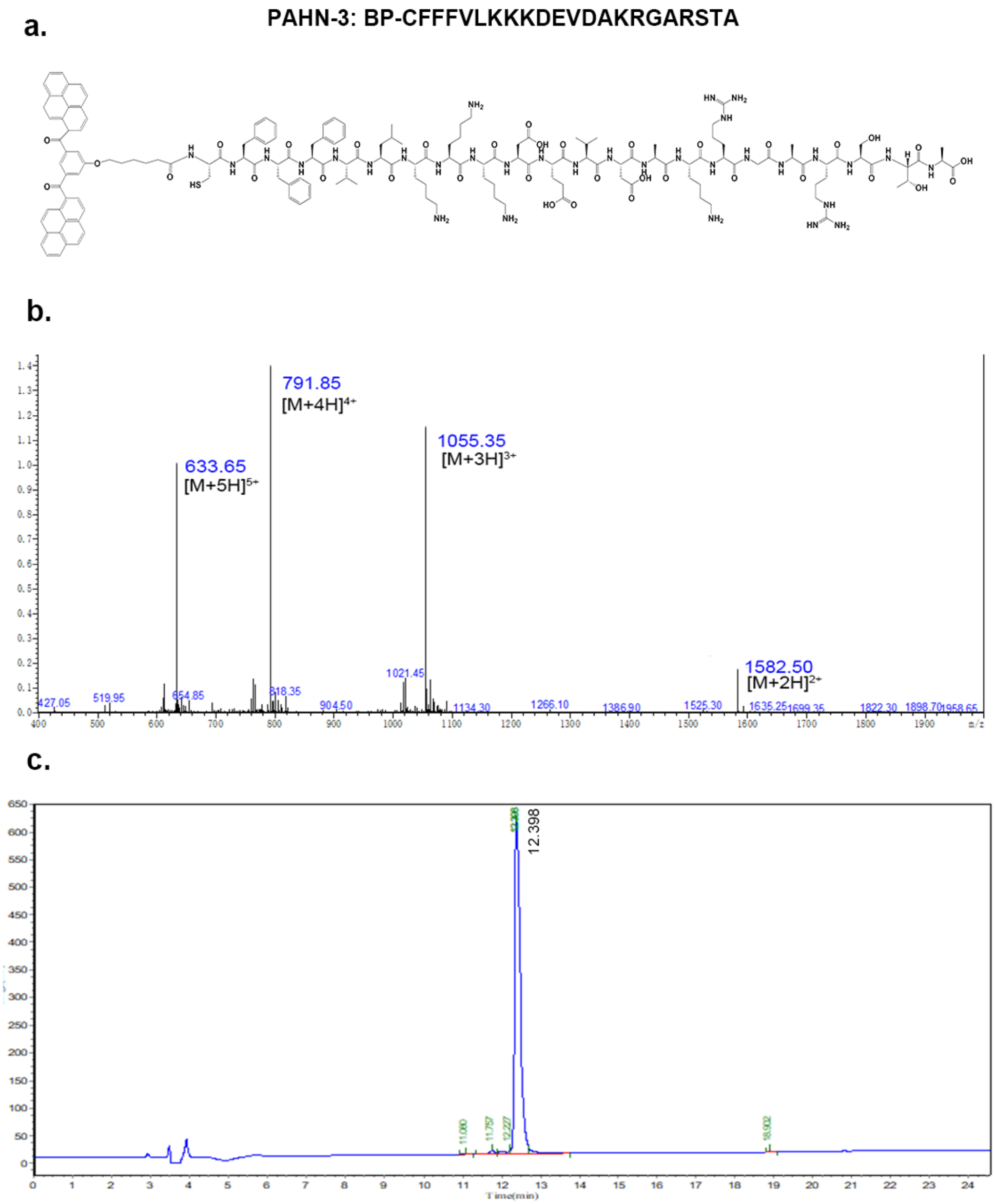

Figure S3. a) The chemical structure, b) mass spectrum and c) HPLC trace of **PAHN-3**. The method of HPLC spectra was as follows: solvent A, 0.1% trifluoroacetic acid in 100% acetonitrile; solvent B, 0.1% trifluoroacetic acid in 100% water; 0.01 min, 85% solvent B 25 min, 47% solvent B.

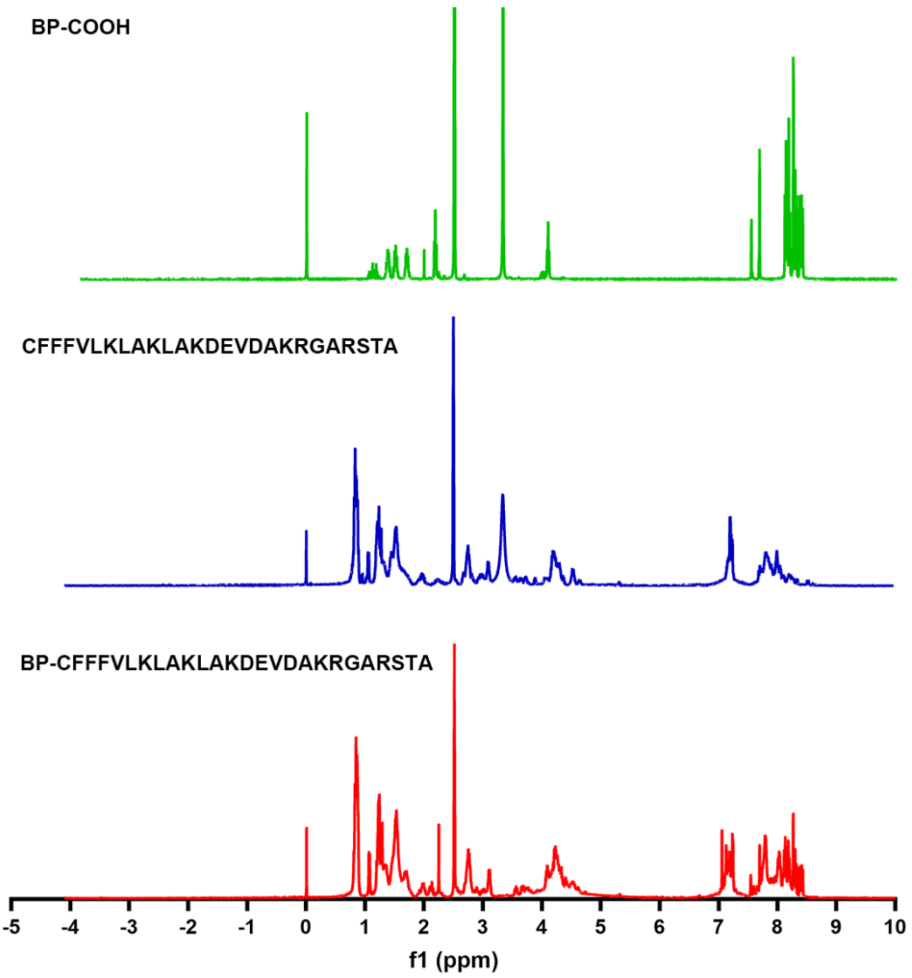

Figure S4. ^{1}HNMR spectrum of BP, polypeptide, and **PAHN-1**

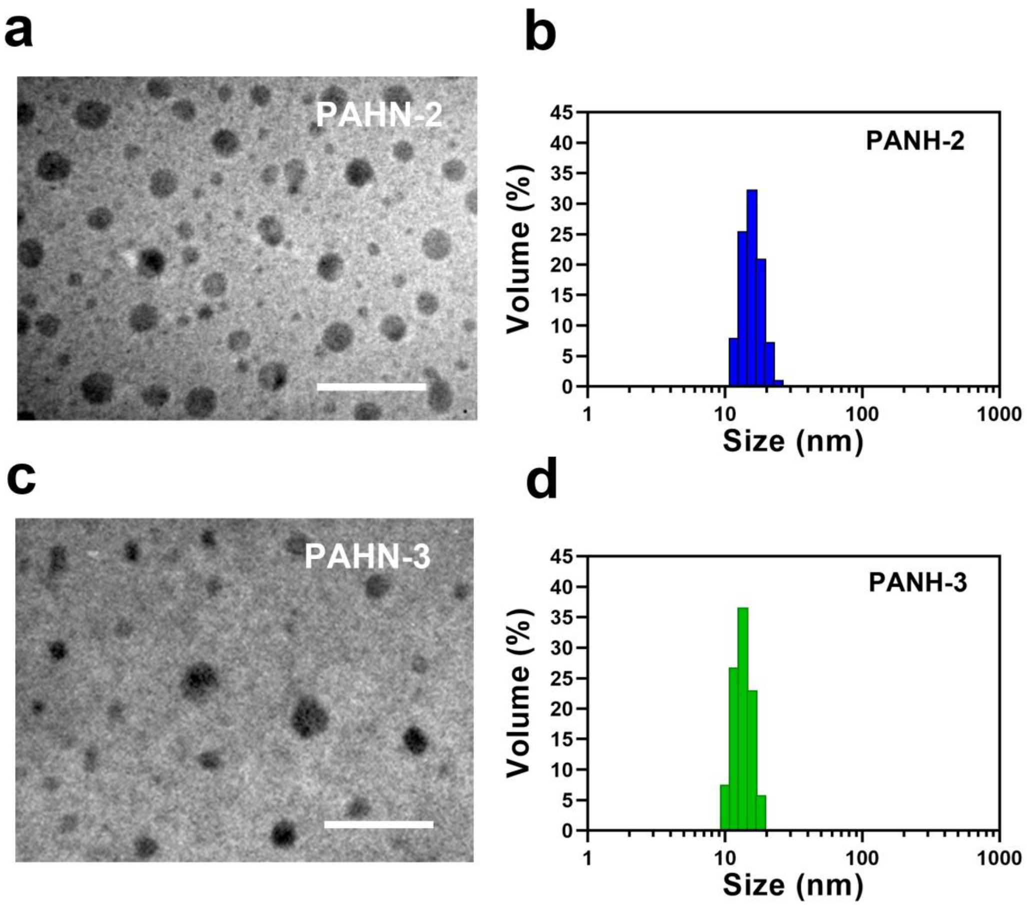

Figure S5. a-b) TEM image and size distribution of **PAHN-2;** c-d) TEM image and size distribution of **PAHN-3.** The scale bars are 50 nm

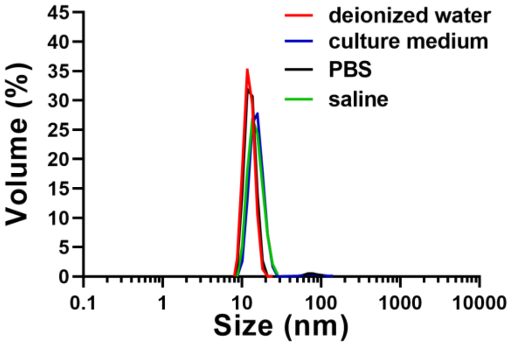

Figure S6. Diameter changes of PAHN-1 in various media

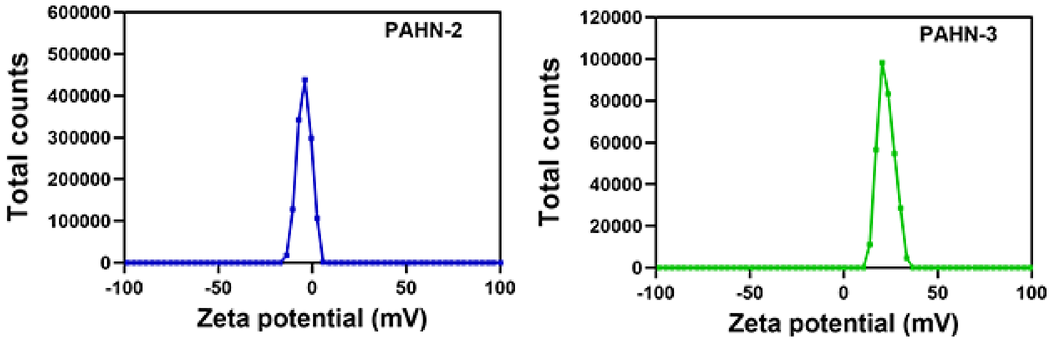

Figure S7. Zeta potentials of **PAHN-2** and **PAHN-3.**

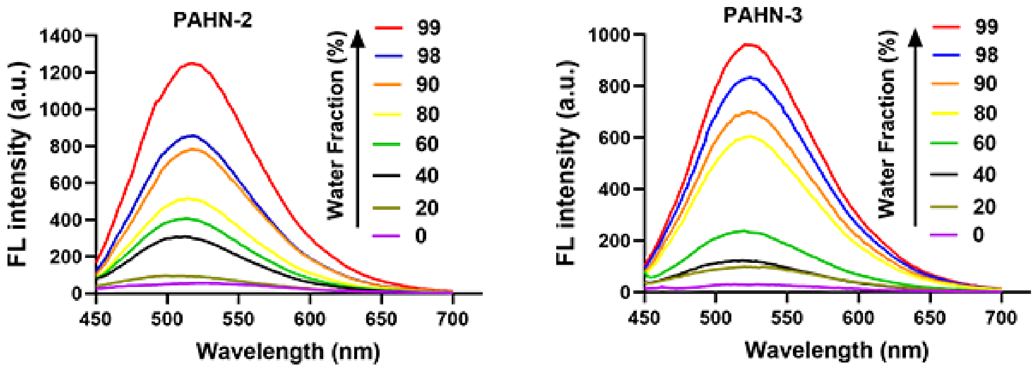

Figure S8. FL intensity changes of **PAHN-2** and **PAHN-3** in the conditions of different water fractions

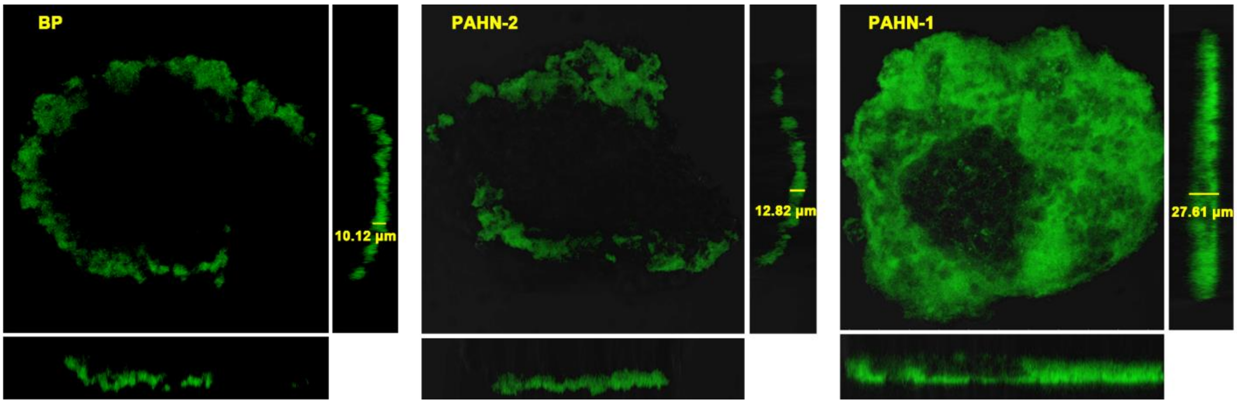

Figure S9. Quantitative analysis of the penetration depth of BP, **PAHN-2** and **PAHN-1** in three-dimensional tumor spheroid models

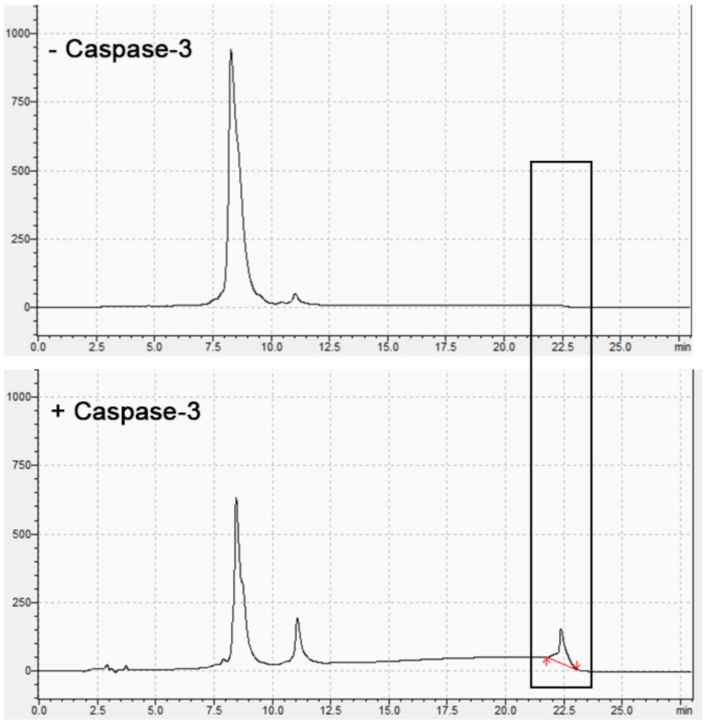

Figure S10. HPLC traces of **PAHN-1** before and after incubating with caspase-3 for 12 h at 37 °C

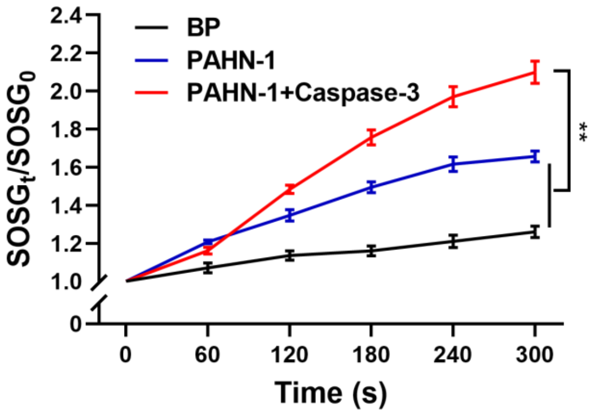

Figure S11. Fluorescence increasing rate of SOSG in BP, **PAHN-1** and **PAHN-1** + caspase-3 under different US irradiation time

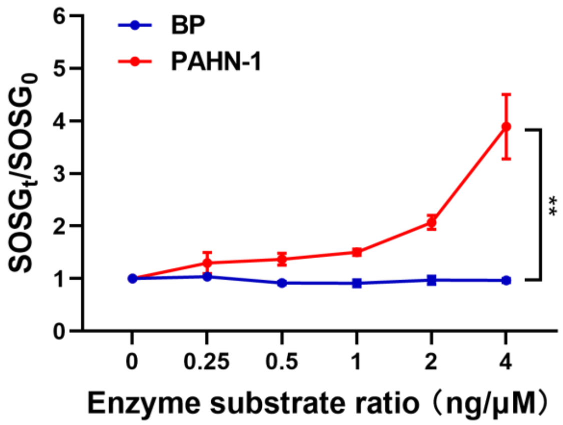

Figure S12. Fluorescence increasing rate of SOSG in BP, **PAHN-1** with various enzyme substrate ratio.

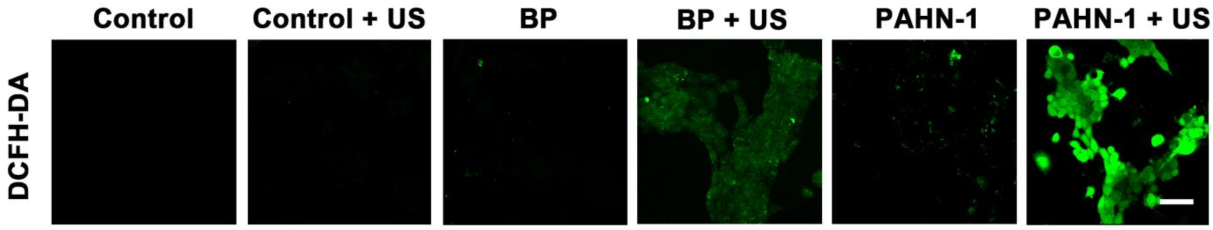

Figure S13. CLSM images of DCFH-DA-stained 4T1 cells subjected to various treatments. The scale bars is 50 μm.

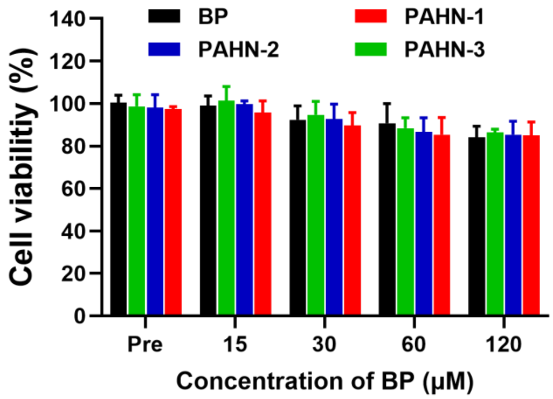

Figure S14. Cytotoxicity of BP, **PAHN-1**, **PAHN-2** and **PAHN-3** against L929 cells

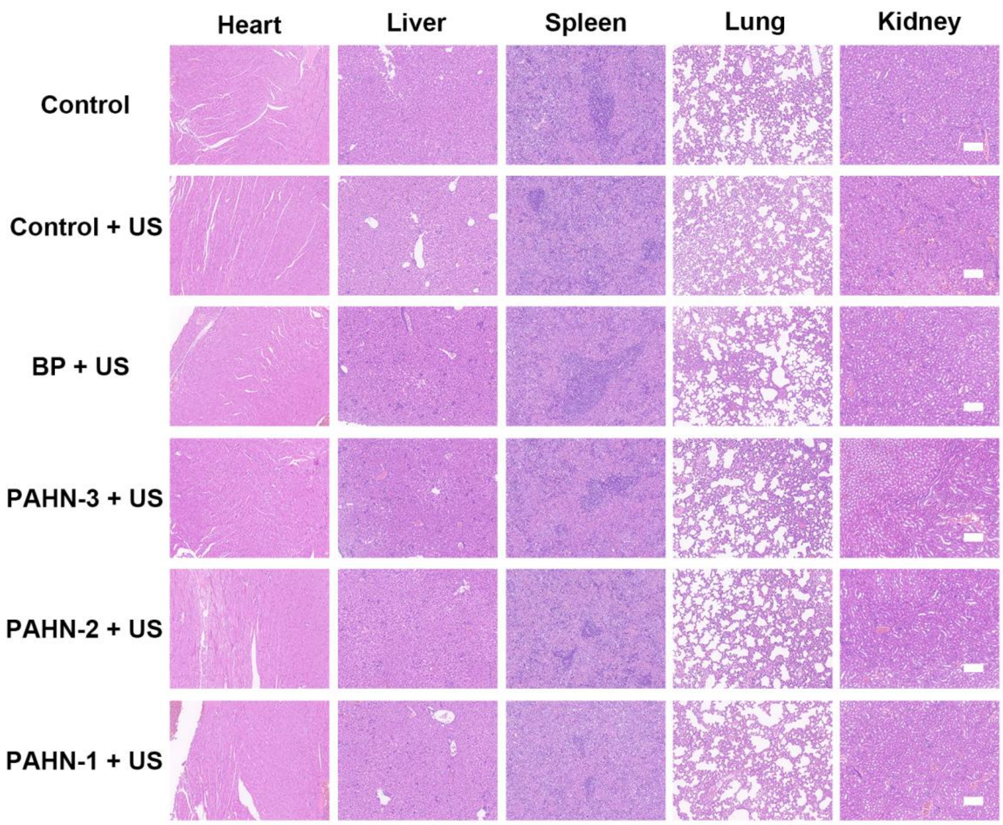

Figure S15. H&E staining of the major organs in mice sacrificed at 16 d after various treatments. The scale bars are 100 μm.

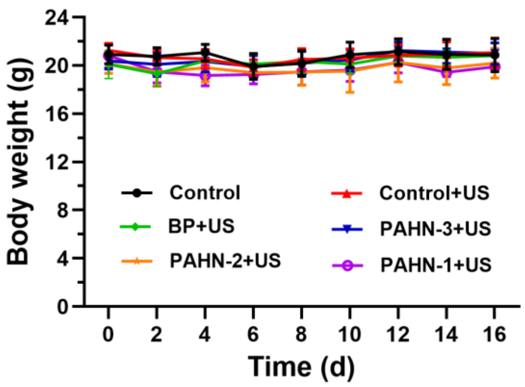

Figure S16. The body weight monitoring of tumor-bearing mice during period of therapy

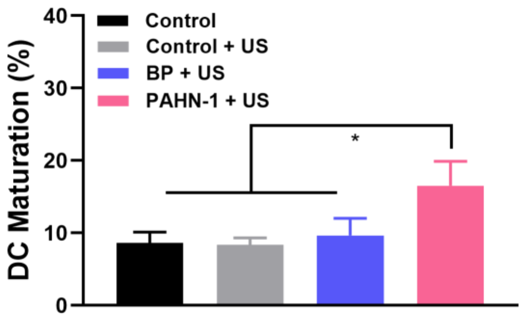

Figure S17. The DC maturation levels in primary tumors of 4T1 tumor-bearing mice after receiving different treatments.

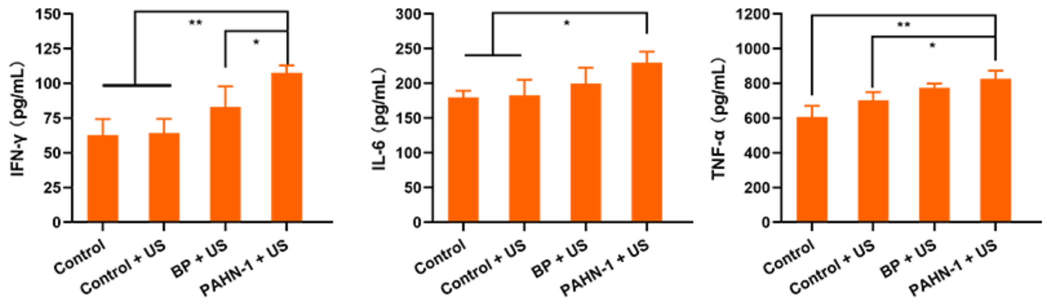

Figure S18. IFN-γ, IL-6 and TNF-α, levels in serum from mice of various groups after different treatments.

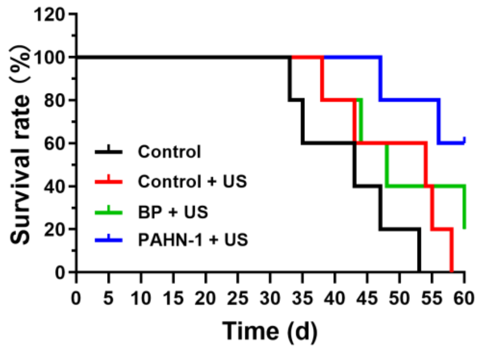

Figure S19. Survival curves of 4T1 tumor-bearing mice in each group during 60-day observation
